# Combination of long-term ^13^CO_2_ labeling and isotopolog profiling allows turnover analysis of photosynthetic pigments in Arabidopsis leaves

**DOI:** 10.1186/s13007-022-00946-3

**Published:** 2022-10-01

**Authors:** Anh Thi-Mai Banh, Björn Thiele, Antonia Chlubek, Thomas Hombach, Einhard Kleist, Shizue Matsubara

**Affiliations:** 1 IBG-2: Plant Sciences, Forschungszentrum Jülich, 52425 Jülich, Germany; 2 IBG-3: Agrosphere, Forschungszentrum Jülich, 52425 Jülich, Germany

**Keywords:** Carotene, Carotenoids, Chlorophyll, Lutein, Pigment metabolism, Stable isotope labeling, Turnover, ^13^CO_2_

## Abstract

**Background:**

Living cells maintain and adjust structural and functional integrity by continual synthesis and degradation of metabolites and macromolecules. The maintenance and adjustment of thylakoid membrane involve turnover of photosynthetic pigments along with subunits of protein complexes. Quantifying their turnover is essential to understand the mechanisms of homeostasis and long-term acclimation of photosynthetic apparatus. Here we report methods combining whole-plant long-term ^13^CO_2_ labeling and liquid chromatography - mass spectrometry (LC–MS) analysis to determine the size of non-labeled population (NLP) of carotenoids and chlorophylls (Chl) in leaf pigment extracts of partially ^13^C-labeled plants.

**Results:**

The labeling chamber enabled parallel ^13^CO_2_ labeling of up to 15 plants of *Arabidopsis thaliana* with real-time environmental monitoring ([CO_2_], light intensity, temperature, relative air humidity and pressure) and recording. No significant difference in growth or photosynthetic pigment composition was found in leaves after 7-d exposure to normal CO_2_ (~ 400 ppm) or ^13^CO_2_ in the labeling chamber, or in ambient air outside the labeling chamber (control). Following chromatographic separation of the pigments and mass peak assignment by high-resolution Fourier-transform ion cyclotron resonance MS, mass spectra of photosynthetic pigments were analyzed by triple quadrupole MS to calculate NLP. The size of NLP remaining after the 7-d ^13^CO_2_ labeling was ~ 10.3% and ~ 11.5% for all-*trans*- and 9-*cis*-β-carotene, ~ 21.9% for lutein, ~ 18.8% for Chl *a* and 33.6% for Chl *b*, highlighting non-uniform turnover of these pigments in thylakoids. Comparable results were obtained in all replicate plants of the ^13^CO_2_ labeling experiment except for three that were showing anthocyanin accumulation and growth impairment due to insufficient water supply (leading to stomatal closure and less ^13^C incorporation).

**Conclusions:**

Our methods allow ^13^CO_2_ labeling and estimation of NLP for photosynthetic pigments with high reproducibility despite potential variations in [^13^CO_2_] between the experiments. The results indicate distinct turnover rates of carotenoids and Chls in thylakoid membrane, which can be investigated in the future by time course experiments. Since ^13^C enrichment can be measured in a range of compounds, long-term ^13^CO_2_ labeling chamber, in combination with appropriate MS methods, facilitates turnover analysis of various metabolites and macromolecules in plants on a time scale of hours to days.

**Supplementary Information:**

The online version contains supplementary material available at 10.1186/s13007-022-00946-3.

## Background

Living cells need maintenance to preserve structural and functional integrity. The maintenance involves breakdown, (re)synthesis and active transport of molecules such as proteins and membrane lipids. While turnover (continual replacement by degradation and synthesis) of macromolecules—proteins in particular—is costly for cells, it allows adjustments of biochemical machinery to environmental changes [1].

Plants have relatively low rates of protein turnover compared to bacteria. Early investigations on protein turnover using ^14^CO_2_ labeling have indicated turnover rate of approx. 0.1 ~ 0.2 d^−1^ in leaves of tobacco, bean, wheat and barley, whereas bacterial cells may have overall protein turnover of 1.2–1.4 d^−1^ [1]. More recently, proteomic studies using ^15^ N labeling have determined degradation rate (K_d_; equivalent to turnover rate in a steady state) of numerous proteins, showing the median of 0.08 d^−1^ and 0.11 d^−1^ in barley and Arabidopsis leaves, respectively [2, 3]. The proteome analysis also highlighted great variations in K_d_ among proteins. In Arabidopsis leaves, in which K_d_ was estimated for 1228 non-redundant proteins, ~ 15% of the proteins had > 0.22 d^−1^ whilst ~ 13% had < 0.055 d^−1^ [3]. Among high-turnover proteins detected in both barley and Arabidopsis are THI1 (1.65 d^−1^ and 1.93 d^−1^ in barley and Arabidopsis, respectively) and THIC (0.64 d^−1^ and 0.89 d^−1^) in thiamine biosynthesis, and D1 protein (0.94 d^−1^ and 1.08 d^−1^) in photosystem II (PSII) reaction center [2, 3]. THI1 and D1 are considered “suicide proteins” because THI1 is a single-use enzyme that serves as a co-substrate by donating a sulfur atom of a cysteine [4, 5] and D1 acts as a safety device that is sacrificed to protect the rest of PSII against photooxidative damage [6–8].

Given the costs of protein turnover, one may ask: are high-turnover proteins worth the benefits? Some attempts have been made to estimate the costs and benefits of D1 damage and repair in oxygenic photosynthesis [9, 10]. The calculation depends on the definition of costs (energy requirement for degradation and synthesis of protein and RNA, additional needs for N and P, missed opportunity for photosynthesis) and the extent of photodamage. The latter, in turn, is influenced by environmental conditions and efficacy of photoprotective mechanisms, including thermal energy dissipation, scavenging of reactive oxygen species, alternative electron transport pathways, state transitions and chloroplast movement [6, 9]. Should it be necessary to replace pigments and lipids in the reaction center and core complex of PSII during the D1 turnover, this would increase the costs of repair and maintenance [9]. A PSII core complex harbors in total 35 chlorophyll *a* (Chl *a*) molecules, two pheophytins, 11 all-*trans*-β-carotenes (β-Car), two plastoquinones, two haem irons and more than 20 lipids besides Mn, Ca, Cl and bicarbonate [11]. We must also keep in mind that not only D1 but also other proteins associated with photosynthesis, such as PetD of cytochrome *b*_6_*f* complex and PIFI of chloroplast NAD(P)H dehydrogenase complex, have relatively high K_d_ values (> 0.5 d^−1^) in Arabidopsis [8].

Previously we have shown the turnover of Chl *a* and all-*trans*-β-Car in leaves of Arabidopsis plants under illumination [12, 13]. A 30-min pulse labeling with ^14^CO_2_ resulted in rapid incorporation of ^14^C in these pigments, whereas Chl *b* and xanthophylls were not labeled throughout the subsequent 10-h chase [12]. Treatment with lincomycin, an inhibitor of plastid translation and thus D1 synthesis, quickly and strongly suppressed ^14^C incorporation in Chl *a*, and to a lesser extent also β-Car, suggesting a link to the D1 turnover [13]. Although detection of radioactive ^14^C is highly sensitive as well as selective, turnover rates could not be determined in these studies due to the unknown number of ^12^C/^14^C substitution per molecule. For example, if all C atoms of Chl *a* and β-Car are labeled with ^14^C, a molecule of Chl *a* (C_55_H_72_O_5_N_4_Mg) will have stronger radioactivity than a β-Car (C_40_H_56_). However, if Chl *a* is partially labeled, let us say 20 C atoms out of 55, its radioactivity is only a half of fully labeled β-Car. Another downside of our ^14^CO_2_ labeling was the use of excised leaves to minimize radioactive wastes, which restricted the duration of the pulse-chase labeling experiments [12, 13].

^13^C offers a good alternative to ^14^C in these respects. ^13^C is a stable, non-hazardous isotope that can be detected and quantified by mass spectrometry (MS). For each compound, mass spectra can show the relative abundance of individual isotopologs having different isotopic compositions (e.g. ^13^C_55_, ^12^C_35_^13^C_20_, ^12^C_55_ etc. for Chls), thus enabling the counting of non-labeled and ^13^C-labeled (fully or partially) molecules. As the first step to study turnover of photosynthetic pigments, we established a liquid chromatography (LC)-MS analysis method for isotopolog profiling of carotenoids. This method, recently described in [14] for lutein (Lut), allows identification and quantification of isotopologs in ^13^C-labeled leaf pigment extracts. Similar methods are also needed for Chls and other carotenoids to analyze turnover of these pigments in a single run. Furthermore, long-term whole-plant ^13^CO_2_ labeling is ideally performed in a chamber, in which the environmental conditions can be controlled and monitored during labeling experiments. Such a chamber will open up new possibilities for turnover studies of many different compounds in plants.

Here we report a protocol of 7-d ^13^CO_2_ labeling in a chamber that was specially designed and constructed for this type of experiments. We also describe the LC–MS analysis methods to identify isotopologs and calculate non-labeled population (NLP) of β-Car, Chl *a* and Chl *b* besides Lut in pigment extracts of ^13^C-labeled leaves. With these methods of ^13^CO_2_ labeling and isotopolog analysis at hand, turnover of photosynthetic pigments and other metabolites can be studied in the future by time course experiments.

## Methods

### Plant material and growth conditions

Plants of *Arabidopsis thaliana* (Columbia-0) were grown in 300-mL plastic cups with lids (Additional file 1: Fig. S1; Bürkle, Bad Bellingen, Germany) which tightly fit into holders of the labeling chamber described below. After cups had been filled with moist soil (Dachstaudensubstrat SoMi 513, Hawita, Vechita, Germany), 1–2 cm^3^ of seed starting soil (Pikier Erde, Blaster Einheitserdewerk, Fröndenberg, Germany) was put in the center of the top soil where individual seeds were sown. Cotyledons grew out of the cup through a hole (3 mm diameter) made in the center of the lid. The lid thus separated the aboveground from root system and soil to minimize the impact of root and soil respiration on the CO_2_ composition inside the labeling chamber. The lid (except the hole) was covered with aluminum foil to suppress algal growth on the soil surface. Two holes (~ 4.5 mm diameter; Additional file 1: Fig. S1) made in the bottom of the cups allowed bottom watering.

Plants were cultivated in a climate chamber under 12 h/12 h light/dark, 23 °C/18 °C air temperature and constant 60% relative air humidity. Illumination was provided by fluorescent tubes (Fluora L36 W/77, Osram, Munich, Germany) which gave light intensity of ~ 100 µmol photons m^−2^ s^−1^ at plant height. Care was taken to keep soil moisture by regular watering from the bottom. Five weeks after sowing, 15 plants of similar projected leaf area (PLA, 14–16 cm^2^) were selected for a preliminary CO_2_ experiment to develop a gas flow rate protocol (see below). Plants were transferred to the labeling chamber installed in a separate climate chamber. After the flow rate protocol had been established, a new batch of plants were cultivated in the same way and 19 plants having 14–16 cm^2^ PLA were selected for a ^13^CO_2_ experiment (day 0 in Additional file 1: Figs. S2, S3a). Of these, 15 were placed in the labeling chamber while the remaining four plants stayed outside the labeling chamber (control).

The rosettes of the 19 plants were individually harvested after seven light/dark cycles in ^13^CO_2_ or ambient air (day 8 in Additional file 1: Figs. S2, S3b). The rosettes were quickly photographed for visual documentation before freezing in liquid N_2_ for pigment analysis.

### Measurement of PLA

During plant cultivation, PLA was determined daily by using the Growscreen-FLUORO method [15] or by taking a top-view image of the plants. In the latter case, a blue reference chip (2 cm diameter) was placed next to the plant (Additional file 1: Figs. S2–S4) to facilitate pixel-to-area conversion. Images were analyzed by ImageJ [16] to obtain PLA.

### ^13^CO_2_ labeling

**Fig. 1:**
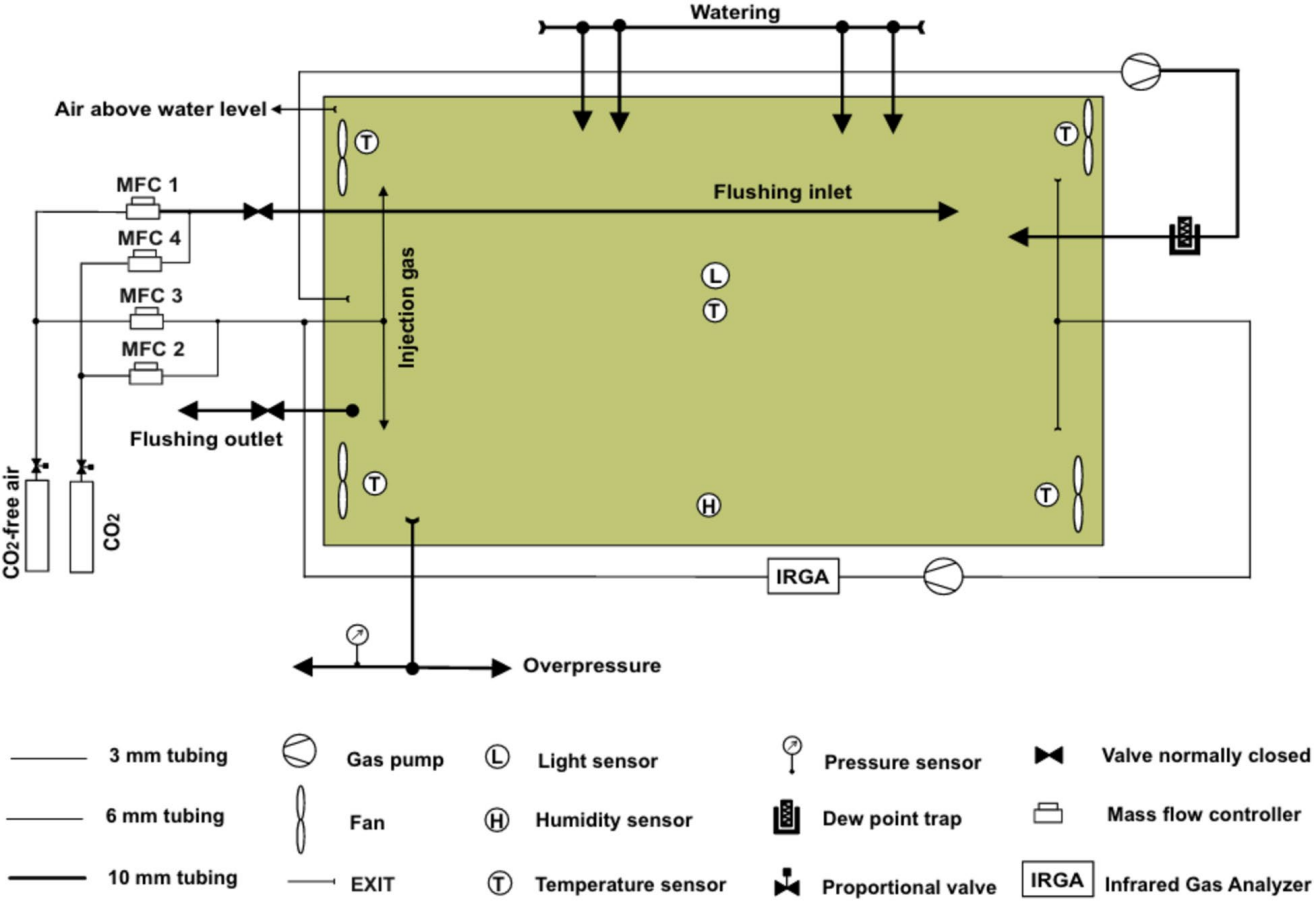
Schematic overview of the ^13^CO_2_ labeling chamber. The devices to control CO_2_ concentration (mass flow controller, MFC) and air humidity (dew point trap) are depicted along with the sensors for CO_2_ (infrared gas analyzer, IRGA), temperature, humidity, light intensity and pressure. The colored background shows the chamber area (top view). The arrows indicate the directions of air (or water) flow. The size of the arrows and the thickness of the lines correspond to the inner diameter of tubing (polytetrafluoroethylene or metal)

We constructed a chamber for long-term ^13^CO_2_ labeling of small plants such as Arabidopsis (Fig. 1). It is equipped with the following control and measuring devices: four mass flow controllers (MFC1–MFC4; EL-FLOW F-201CV-10 K-RAR-00-V, F-200C-RFB-33-Z, F-201C-RFB-33-V, F-201CV-100-RAR-00-Z, Bronkhorst Deutschland Nord, Kamen, Germany) for CO_2_-free air and CO_2_, an infrared gas analyzer (IRGA; LI-840, LI-COR, Lincoln, NE, USA) to measure [CO_2_], a custom-made dew point trap cooled by a water bath (6 °C; Julabo F32 MA, JULABO Labortechnik, Seelbach, Germany) to reduce air humidity, four fans (8412 NGMV, EBM papst, Mulfingen, Germany) in four corners to mix the air inside the chamber, five temperature sensors (type K, mawi-therm Temperatur-Prozeßtechnik, Essen, Germany) placed in four corners and at the center, and one sensor each for air humidity (DKRF400, Driesen + Kern, Bad Bramstedt, Germany), light intensity (LI-190R, LI-COR) and pressure (M260 Multisense, Setra Systems, Boxborough, MA, USA).

**Fig. 2:**
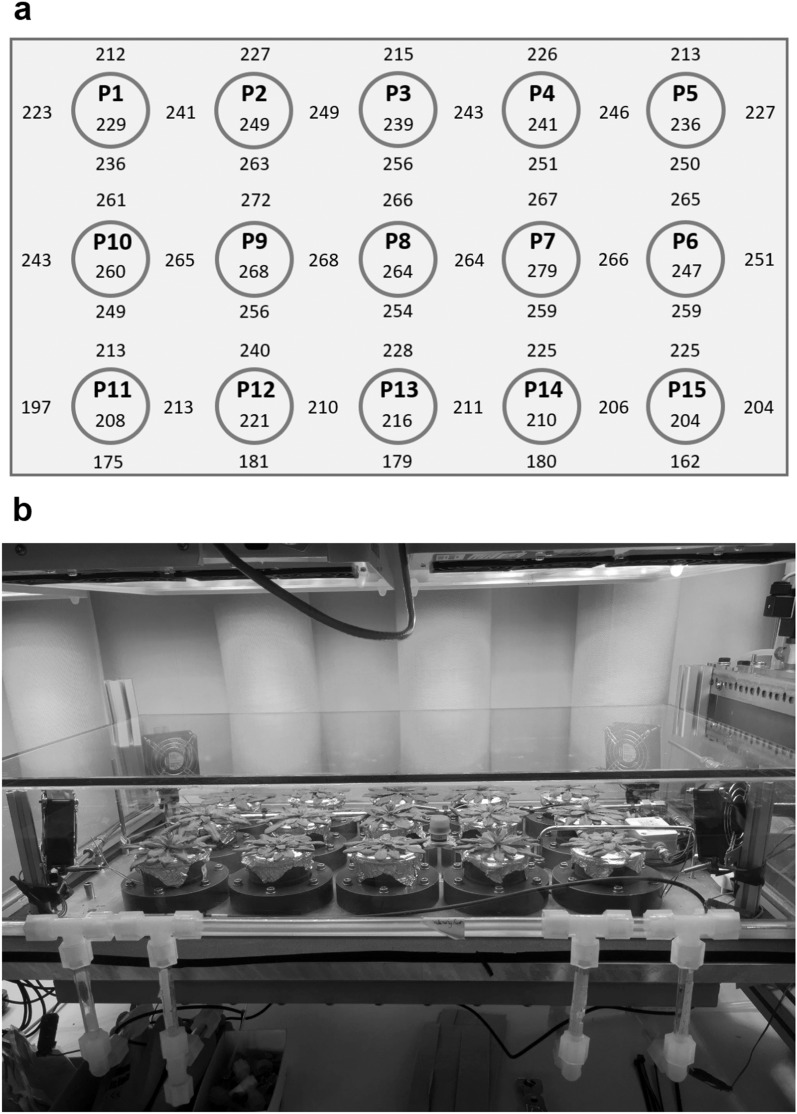
Plant positions in the labeling chamber. **a** The positions of 15 plants (P1–P15) and the light intensity (in µmol photon m^−2^ s^−1^) distribution measured in and around each plant position without the glass cover of the labeling chamber. The light intensity thus measured was ranging between 204 (P15) and 279 (P7) µmol photon m^−2^ s^−1^ among the 15 positions, with the mean intensity of 238 µmol photon m^−2^ s^−1^. **b** A picture of the closed labeling chamber with 15 Arabidopsis plants placed under LED lamps in a controlled climate chamber. The bottom of the plant cups (see Additional file 1: Fig. S1 for description of the plant cup) was touching the water in a shallow basin attached to the lower surface of the chamber body. The basin can be filled and drained through watering tubes (seen in the front) without opening the chamber

The labeling chamber has 15 airtight holders for plant cups described above (Fig. 2a). The chamber can be closed with a glass cover (50 × 88 × 16 cm; L × W × H) (Fig. 2b). The connection between the glass cover and the chamber body is sealed with polyurethane foam gaskets (Armaflex AF/E 10 mm, Armacell, Münster, Germany). As this sealing is not airtight, a small overpressure is needed inside the labeling chamber to prevent diffusion of ambient air from the outside (see below). The overpressure results in air leakage and thus a loss of ^13^CO_2_ from the chamber through the glass cover sealing. A shallow plastic basin attached to the lower surface of the chamber body can be filled with water through a tubing (Fig. 2b) without opening the chamber. When plant cups are put in the holders, the bottom of the cups touches the water in the basin, thus allowing bottom watering.

While the labeling chamber is in operation, two gas pumps (NMP830KVDCB and N 816 K_DC-B, KNF Neuberger, Freiburg, Germany) continuously circulate the internal air through bypasses that lead to LI-840 and the dew point trap (Fig. 1). The flow rates of the gas pumps were set to 1 L min^−1^ (to LI-840) and 14 L min^−1^ (to the dew point trap). The four MFCs are controlled by a custom-made computer program (made with LabVIEW 2014; National Instruments, Austin, TX, USA), which also visualizes environmental readings of the sensors in real time and records the data every minute. The flow rates of MFC2 (CO_2_ or ^13^CO_2_) and MFC3 (CO_2_-free air prepared by filtering the ambient air through an industrial adsorption dryer KEN-MT 3800 MSTE; Parker Hannifin, Kaarst, Germany) were adjusted such that [CO_2_] of ~ 400 ppm was maintained during the light period (Additional file 1: Fig. S5a–c). The mass flow of CO_2_-free air (1 L min^−1^ or 2 L min^−1^) created a small overpressure (~ 60 Pa or 160–170 Pa; Additional file 1: Fig. S5d) inside the labeling chamber compared to the outside (atmospheric pressure, ~ 101.3 kPa) to prevent diffusion of external air into the chamber. The amount of CO_2_ injection was increased as the light intensity increased in the morning (see below for light intensity regime) and as the plants grew larger (Additional file 1: Fig. S5b). To keep daytime [CO_2_] constant, the amount of CO_2_ injection must be balanced with net CO_2_ fixation of the plants, which depends on the genotype, size (leaf area) and development, as well as the conditions during cultivation and labeling.

We did not inject CO_2_ during the dark period (except during 20-min equilibration immediately before the onset of light period; see below) while the flow of CO_2_-free air was increased from 1 L min^−1^ to 2 L min^−1^ (Additional file 1: Fig. S5a, b) to minimize accumulation of respired CO_2_ with unknown degree of ^13^C labeling. As a result, nocturnal [CO_2_] decreased to below 100 ppm (Additional file 1: Fig. S5c). Since plants were grown in the ambient air, we set the flow rate of CO_2_-free air to 2 L min^−1^ during the first light period when light respiration of ^12^CO_2_ was expected to dilute ^13^CO_2_. The flow rate of CO_2_-free air was reduced to 1 L min^−1^ during the subsequent light periods (presumably decreasing ^12^CO_2_ in respiration) to lower the overpressure and thus reduce the loss of ^13^CO_2_ from the labeling chamber. During the dark periods, the high flow rate of CO_2_-free air and thus the high overpressure (Additional file 1: Fig. S5d) effectively blocked the diffusion of external air into the chamber. Should the overpressure exceed a given threshold (250 Pa in this study), a safety valve would open automatically to release the air from the chamber. This threshold, however, was never reached during the experiments (Additional file 1: Fig. S5d). In addition to the high flow rate during the dark periods, the labeling chamber was flushed with CO_2_-free air (MFC1; 10 L min^−1^ for 45 min) at the end of each dark period to get rid of nocturnal air containing respiratory CO_2_ of unknown isotopic composition. The flushing was followed by 20-min equilibration with a fresh mixture of CO_2_ (MFC4; 5 mL min^−1^) and CO_2_-free air (MFC1; 10 L min^−1^) immediately before the onset of the light period. An outlet valve was automatically opened during flushing and equilibration to keep the overpressure low.

We conducted a preliminary experiment using normal CO_2_ (purchased from Air Products, Hattingen, Germany) to establish a flow rate protocol (Additional file 1: Figs. S2–S6). This protocol was then used in the experiment with ^13^CO_2_ (> 99 atom %; Linde, Pullach, Germany). Since LI-840 has a very low sensitivity to ^13^CO_2_ [17], the readings were low in the ^13^CO_2_ experiment (Additional file 1: Fig. S6). Nevertheless, light/dark patterns of [CO_2_] were highly reproducible in both CO_2_ and ^13^CO_2_ conditions, suggesting that the flow rate protocol provided similar [CO_2_] inside the labeling chamber. It should be noted, however, that [CO_2_] and [^13^CO_2_] may vary if the size and growth rate (and thus the total CO_2_ assimilation) of the plants differ between the CO_2_ and ^13^CO_2_ experiments due to suboptimal selection of plants or occurrence of stressed plants (see below).

The labeling chamber was placed under LED lamps (L4A Series 10, HelioSpectra, Göteborg, Germany) in the climate chamber running with 12 h/12 h light/dark and constant 20 °C and 60% relative air humidity. The intensity of LED lamps was increased or decreased in three steps at the beginning or at the end of the 12-h light period, respectively (Additional file 1: Fig. S7a). The light period started with 30-min dim light (~ 28 μmol photons m^−2^ s^−1^), followed by an increase to ~ 115 μmol photons m^−2^ s^−1^ (60 min) and then to ~ 200 μmol photons m^−2^ s^−1^ (9 h) before returning to darkness in the reverse sequence. The light intensity shown in Additional file 1: Fig. S7a was measured by LI-190R mounted near the plant position P7 and P8 (see Figs. 1, 2) in the closed chamber (i.e., under the glass cover). Figure 2a illustrates light distribution in the open chamber measured at plant height using X1 optometer (Gigahertz-Optik, Türkenfeld, Germany). The mean intensity of the 15 plant positions was 238 μmol photons m^−2^ s^−1^ ± 10% SD without the glass cover. Comparing the values measured at around P7 and P8 in the open and closed chamber, the glass cover apparently reduced the light intensity by ca. 25%. For the control plants outside the labeling chamber, the intensity of LED was set to the level similar to the condition in Additional file 1: Fig. S7a.

While the climate chamber had constant 20 °C and 60% relative air humidity, illumination and plant transpiration raised the air temperature and humidity inside the closed labeling chamber (Additional file 1: Fig. S7b, c). The air temperature increased to ~ 23 °C during the light period and decreased to ~ 20.5 °C during the dark period. Although the chamber has a dew point trap (Fig. 1), the capacity of the dew point trap was not enough to fully compensate for the transpiration of 15 growing plants of Arabidopsis; the air humidity gradually increased from ~ 55% to ~ 75% during the 7-d experiments even though the chamber was flushed daily and the flow rate of CO_2_-free air was kept at 2 L min^−1^ during the dark periods.

The ^13^CO_2_ labeling was stopped after seven light/dark cycles. The chamber was opened in the dim light at the beginning of the light period of day 8 to harvest the whole rosette of each plant. As opening the chamber inevitably exposes plants to ambient air, we collected all plants (also the control) during the 30-min dim-light period to minimize photosynthetic CO_2_ fixation. The total amount of ^13^CO_2_ used by the flow rate protocol (including daily flushing, equilibration and loss in addition to CO_2_ assimilation of 15 plants) during the 7-d labeling was ca. 6.8 L.

### Pigment extraction

The whole rosettes of Arabidopsis plants were individually frozen in liquid N_2_ and ground to powder using pre-cooled mortar and pestle. They were stored at −80 °C until pigment extraction.

About 40 or 80 mg of frozen leaf powder were quickly weighed with an analytical balance (Explorer Pro, OHAUS, Nänikon, Switzerland). Pigments were extracted by using the protocol described before [14]. The weighed frozen leaf powder was ground in 2 mL of chilled acetone under dim light and the homogenate was collected in a 2-mL reaction tube. After 5-min centrifugation at 16,100 rcf (5415D, Eppendorf, Wesseling-Berzdorf, Germany), the supernatant was filtered through a syringe filter (0.45 μm, Chromafil® AO-45/3, Macherey–Nagel, Düren, Germany) into a brown glass vial. All extracts were prepared shortly before injection into LC–MS instruments.

### MS analysis

The LC–MS system consists of a Waters ACQUITY UPLC system and a Waters Xevo TQ-S triple quadrupole MS (hereafter TQ-MS). The LC-Fourier-transform ion cyclotron resonance-MS (hereafter FTICR-MS) consists of an Agilent 1200 series HPLC system and a hybrid linear ion trap FTICR-MS (LTQ FT Ultra, Thermo Fisher Scientific) equipped with a 7 Tesla magnet. Soft ionization was performed in positive ion mode by electrospray ionization (ESI in TQ-MS) or atmospheric pressure chemical ionization (APCI in FTICR-MS). For information about the instrument settings, see [14].

Chromatographic separation was done by a C30 silica column (ProntoSil 200–3-C30, 250 × 4.6 mm, 3 µm, Bischoff Chromatography, Leonberg, Germany) using the method described in [14]. Pigments were identified based on absorption spectra. Chromatograms were extracted at 440 nm and peak area integration was performed by MassLynx software (version 4.1, Waters) for TQ-MS data. To determine pigment concentration, the LC of the TQ-MS system was calibrated with pigment standards purchased from DHI LAB products (Hørsholm, Denmark): Lut, all-*trans*-α-Car, all-*trans*-β-Car, Chl *a* and Chl *b* as well as 9-*cis*-neoxanthin (Neo), violaxanthin (Vio), antheraxanthin (Anthera) and zeaxanthin (Zea). Carotenoid levels relative to Chl (Chl *a* + Chl *b*) were calculated in mmol mol^−1^ Chl. The de-epoxidation state (DES) of the xanthophyll-cycle pigments was defined as (Anthera + Zea) / (Vio + Anthera + Zea).

Mass spectra were obtained at the maximal intensity of the pigment peaks in ion chromatograms (FTICR-MS) or by manually selecting the regions around the maximal peak intensity (TQ-MS) using a full scan mode to cover mass-to-charge ratio (*m*/*z*) between 350 and 1000. Data were processed with MassLynx for TQ-MS and Xcalibur (version 2.0.7, Thermo Fisher Scientific) for FTICR-MS. We first analyzed one each of the ^13^C-labeled and non-labeled (control) samples using both TQ-MS and FTICR-MS to assign mass peaks. The high mass accuracy of FTICR-MS allows assignment of mass peaks to distinct empirical formulae. The resolving power (full width at half maximum) of FTICR-MS was 100,000 at *m*/*z* 400. Based on the peak assignment of FTICR-MS, matching peaks were identified in the corresponding data of TQ-MS. All other samples were analyzed by TQ-MS using the same peak selection schemes.

### Degree of ^13^C labeling (DoL) and NLP of pigments

The base peak intensity (BPI) was calculated for each pigment isotopolog as follows [14]:

1 $${BPI}_{i}=\frac{{I}_{i}}{{I}_{(max) }}\cdot 100$$

where BPI_*i*_ is the base peak intensity of an isotopolog with *i* ^13^C atoms, *i* the number of ^13^C atoms in the isotopolog, I_*i*_ the peak intensity of an isotopolog with *i* ^13^C atoms, and I_(max)_ the highest peak intensity of all isotopologs of the pigment.

The BPI_*i*_ values were then normalized to the sum of BPI_*i*_ of all isotopologs of the pigment.

2 $${BPI}_{i(norm)}=\frac{{BPI}_{i}}{\sum_{i=0}^{n}{BPI}_{i}}\cdot 100$$

BPI_*i*(norm)_ is the normalized base peak intensity of an isotopolog with *i* ^13^C atoms and *n* is the number of C atoms in the pigment molecule (40 for carotenoids, 55 for Chls).

The DoL was calculated for each isotopolog as follows:

3 $${DoL}_{i}=\frac{{BPI}_{i(norm)}\cdot i}{n}$$

where DoL_*i*_ is the degree of ^13^C labeling of an isotopolog with *i* ^13^C atoms.

The overall DoL (ΣDoL) of the pigment can be obtained by adding up DoL_*i*_ of all isotopologs.

4 $$\Sigma DoL=\sum_{i=0}^{n}{DoL}_{i}$$

We estimated the relative abundance of non-labeled pigment population (NLP), which can be defined as the sum of BPI_*i*(norm)_ of all non-labeled isotopologs. Due to natural abundance of ^13^C in the atmosphere (1.1%), isotopologs having a few ^13^C atoms are always found in plant extracts (Additional file 1: Fig. S8). These naturally ^13^C-containing isotopologs were considered "non-labeled".

### Spike test of TQ-MS

Pigment recovery of the TQ-MS system was checked by spike tests. About 40 mg of frozen leaf powder of a ^13^C-labeled Arabidopsis plant were homogenized in 2 mL of chilled acetone. The homogenate was then divided into two aliquots and 0.5 mL of pigment standards (Lut, all-*trans*-β-Car and Chl *a*; all from DHI LAB products) of known concentrations were spiked in one aliquot. Both aliquots were centrifuged and the supernatants filtered into brown glass vials as described above. Following TQ-MS analysis of the pigment standards and the ^13^C-labeled Arabidopsis leaf pigment extract with and without spike (Additional file 1: Fig. S9), BPI_*i*(norm)_ was calculated for the pigments using Eq. 2. Because addition of non-labeled standards should increase the relative abundance of non-labeled isotopologs in the spiked sample, recovery of the added standards can be estimated from the ratio between the measured and the expected increase in NLP of the spiked sample compared to the non-spiked sample.

5 $$Recovery=\frac{measured \,NLP\, increase}{expected \,NLP \,increase}$$

The results of the spike tests are documented in Additional file 2: Table S1. The recovery was 95% or higher for all three pigments.

### Reproducibility of ΣDoL and NLP

Technical reproducibility of ΣDoL and NLP was verified by repeated injections of a ^13^C-labeled Arabidopsis pigment extract into TQ-MS and analyzing ΣDoL and NLP in these data. The mean values (± SD with n = 3 for all-*trans*-βCar, Lut and Chl *a*, n = 4 for Chl *b*) of ΣDoL and NLP thus obtained for each pigment are summarized in Additional file 2: Table S2.

## Results

### ^13^CO_2_ treatment in the labeling chamber

The labeling chamber was first run with normal CO_2_ to establish a flow rate protocol of CO_2_ and CO_2_-free air (see the description in Methods). This was necessary because LI-840, which was connected to the labeling chamber (Fig. 1), measures [CO_2_] at the wavelength of ~ 4.26 μm where ^12^CO_2_ absorbs much more strongly than ^13^CO_2_ (Additional file 1: Fig. S6) [17]. Subsequently, the ^13^CO_2_ labeling experiment was conducted using the same protocol.

Additional file 1: Fig.S2 shows top-view images of the plants at the beginning (day 0) and at the end of the experiment (day 8). Grown in the same conditions, the plants with similar PLA looked very much alike on day 0. After seven light/dark cycles in the normal CO_2_ or ^13^CO_2_ conditions inside the labeling chamber, the plants were visually indistinguishable from the control plants that stayed in the ambient air outside the labeling chamber. The chamber has 15 plant cup holders for simultaneous labeling (Fig. 2). All 15 plants of the ^13^CO_2_ experiment are shown in Additional file 1: Fig.S3. Three plants (P6, P13 and P15) grew less and showed anthocyanin accumulation during the experiment (Fig. 2b); their plastic cups were not placed deep enough in the holders, resulting in suboptimal watering and stomatal closure, and thus less CO_2_ fixation and growth. No sign of stress was recognized in other 12 plants. For comparison, the pictures of 15 plants used in the preliminary experiment with normal CO_2_ are shown in Additional file 1: Fig.S4. Two plants (P9 and P11) were somewhat smaller at the end of the preliminary experiment. Overall, however, growth and visual phenotype of the plants were comparable between the ^13^CO_2_ and normal CO_2_ experiments.

**Fig. 3:**
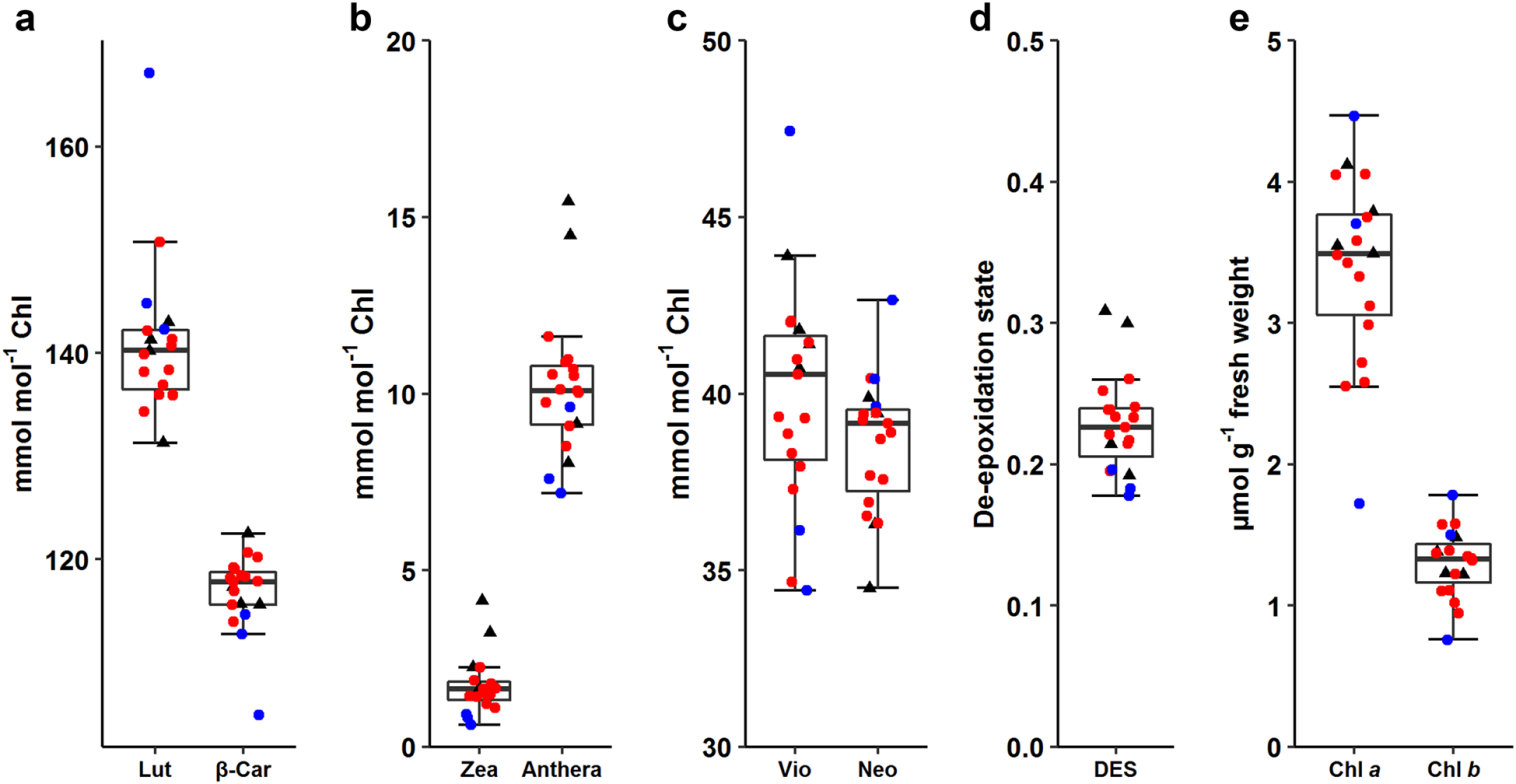
Concentrations of photosynthetic pigments in Arabidopsis plants harvested in the early morning of day 8. **a** Lutein (Lut) and all-*trans*-β-carotene (β-Car). **b** Zeaxanthin (Zea) and antheraxanthin (Anthera). **c** Violaxanthin (Vio) and neoxanthin (Neo). Carotenoid levels relative to the total chlorophyll content (mmol mol^−1^ Chl) are shown. **d** De-epoxidation state (DES) of the xanthophyll cycle calculated as (Anthera + Zea)/(Vio + Anthera + Zea). **e** Chlorophyll *a* (Chl *a*) and chlorophyll *b* (Chl *b*) contents per unit leaf mass (μmol g^−1^ fresh weight). Black triangles represent control plants (*n* = 4) that stayed in the ambient air outside the labeling chamber. For ^13^C-labeled samples, red and blue symbols are for plants with higher (*n* = 12) or lower (*n* = 3) ^13^C incorporation in the pigments, respectively. The latter showed visible stress symptoms (see Additional file 1: Fig. S3b for images of the plants). The box plots are based on all data. The thick horizontal line inside the box shows the median. The middle 50% of the data fall between the upper and lower end of the box. Data beyond the whisker boundaries are outliers

Leaf pigment composition was analyzed in the 15 plants of the ^13^CO_2_ experiment as well as four control plants. The ^13^C-labeled plants and non-labeled control were comparable regarding leaf carotenoid and Chl contents (Fig. 3), except that two control plants had somewhat higher DES of the xanthophyll-cycle pigments (Fig. 3d). Since the leaves were harvested under dim light in the morning, all samples had high levels of Vio, some Anthera and only trace amounts of Zea (Fig. 3b, c). The pigment composition did not systematically differ between the stressed plants (P6, P13 and P15) and the rest.

### Mass peak assignment and calculation of DoL and NLP

The substitution of ^12^C (mass 12.000000) by ^13^C (mass 13.003355) and vice versa can be detected in molecules by MS analysis. In order to identify pigment isotopologs in mass spectra of ^13^C-labeled samples, mass peaks were assigned to empirical formulae by high-resolution FTICR-MS. Matching peaks were then selected in the corresponding data of TQ-MS. Since mass peak assignment of Lut is explained elsewhere [14], we describe below the procedures of peak assignment and analysis focusing on β-Car and Chls. We could not analyze mass spectra of Zea due to the low concentrations in the samples (Fig. 3b); peak assignment of Vio, Anthera and Zea is outside the scope of this study. The mass peak assignment of Lut, however, can be considered a proof of principle for these xanthophylls. The MS analysis of Neo was confronted by co-eluting compounds.

**Fig. 4:**
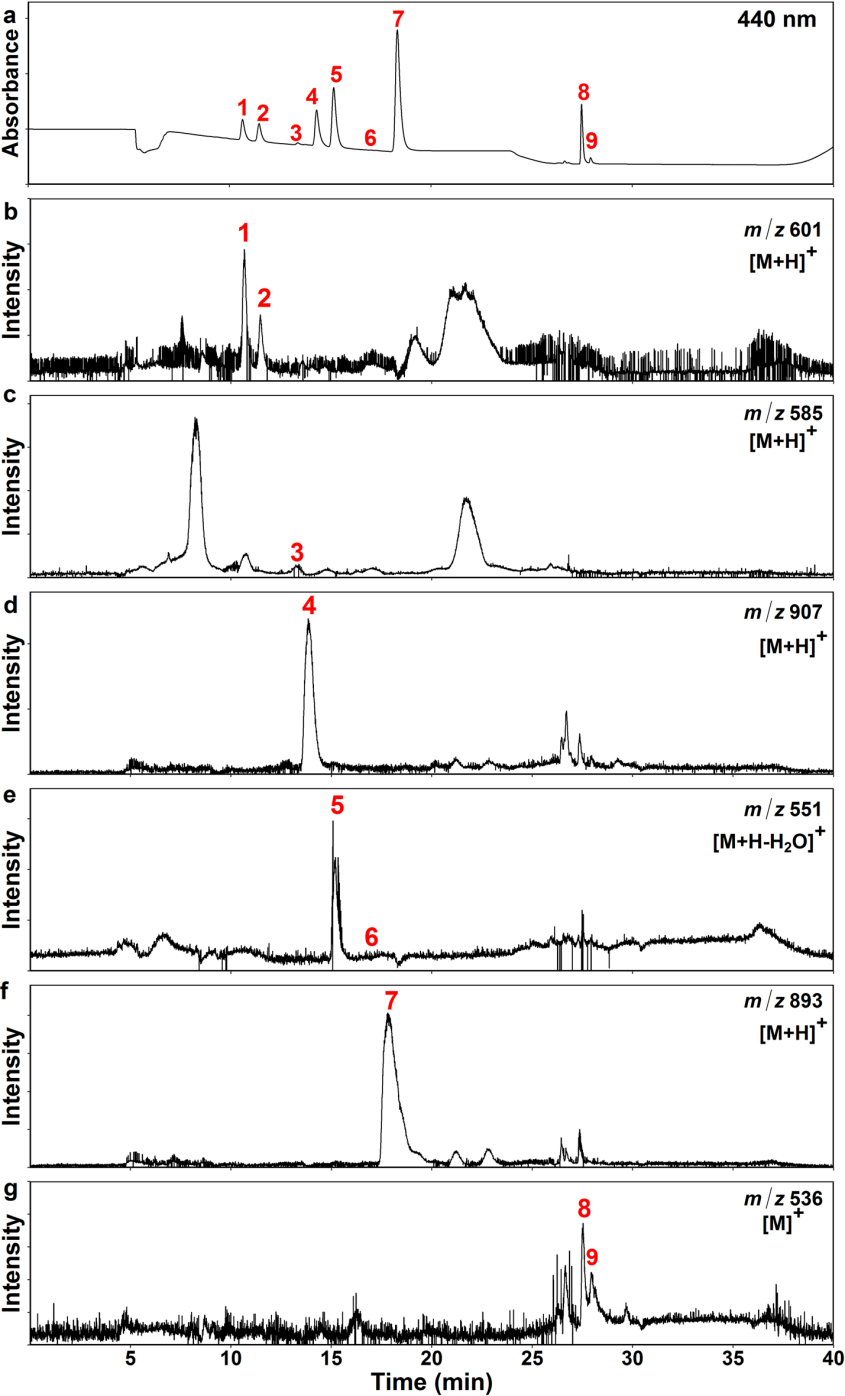
Chromatograms of a ^13^C-labeled Arabidopsis leaf pigment sample obtained by LC-TQ-MS. **a** Pigment separation monitored at 440 nm. The pigment peaks are numbered as follows: 1, Vio; 2, 9-*cis*-Neo; 3, Anthera; 4, Chl *b*; 5, Lut; 6, Zea (if present); 7, Chl *a*; 8, all-*trans*-β-Car; 9, 9-*cis*-β-Car. This sample had a very small amount of Anthera and hardly any Zea. In the same sample, selected ions were monitored at specific mass-to-charge (*m*/*z*) values: **b** Vio and 9-*cis*-Neo; **c** Anthera; **d** Chl *b*; **e** Lut (and Zea if present); **f** Chl *a*; **g** all-*trans*- and 9-*cis*-β-Car. Xanthophylls, especially Lut, tend to lose water upon protonation in positive ion mode

Chromatographic separation of photosynthetic pigments was monitored at 440 nm (Fig. 4a). The ion chromatograms presented in Fig. 4b–g were extracted at nominal mass of monoisotopic pigment ions: ^12^C_40_ for carotenoids and ^12^C_55_ for Chls. Since ionization occurred in positive mode, [M + H]^+^ was the major quasi-molecular ion of Vio (Fig. 4b) and Chls (Fig. 4d, f). Lut has strong tendency to lose water upon protonation [14, 18, 19], forming [M + H–H_2_O]^+^ as the main quasi-molecular ion (Fig. 4e). Vio also gave rise to multiple dehydration products (–H_2_O, –2H_2_O or –3H_2_O) but [M + H]^+^ was still the most abundant ion. In contrast to xanthophylls, both all-*trans* and 9-*cis* isomers of β-Car predominantly formed [M]^+^ in TQ-MS (Fig. 4g) and [M + H]^+^ in FTICR-MS (see below).

**Fig. 5:**
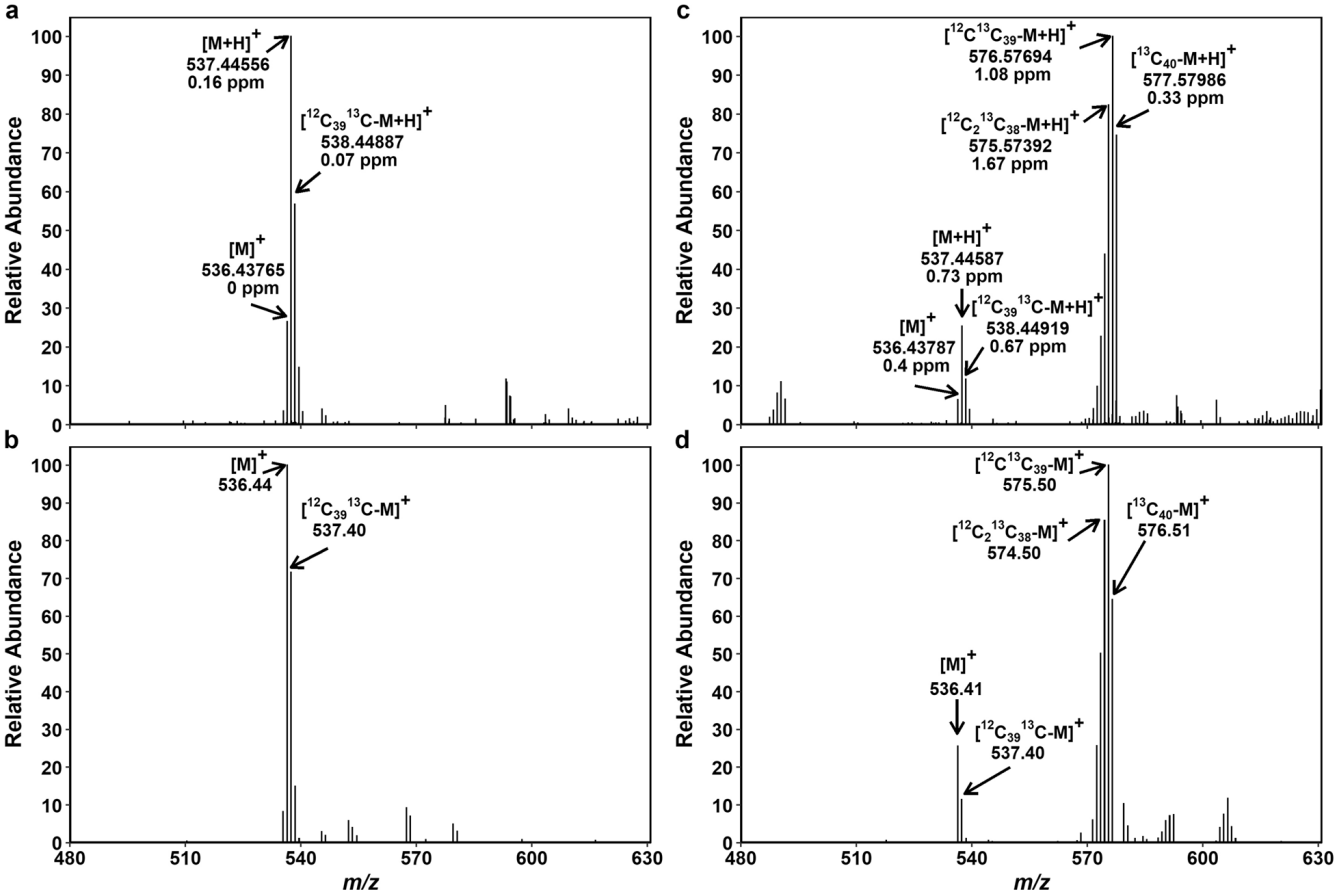
Mass spectra of all-*trans*-β-Car extracted from non-labeled and ^13^C-labeled Arabidopsis plants. FTICR-MS showing two types of quasi-molecular ions of β-Car, [M]^+^ and [M + H]^+^, in a non-labeled (**a**) and a ^13^C-labeled (**c**) sample. Deviations from the expected mass (Δ) are given in parts per million (ppm). TQ-MS in the same non-labeled (**b**) and ^13^C-labeled (**d**) samples as in **a** and **c**. Overlapping mass peaks of [M]^+^ and [M + H]^+^ ions are regarded as [M + H]^+^ or [M]^+^ in the analysis of FTICR-MS and TQ-MS, respectively. Peak assignment of these data is summarized in Additional file 2: Tables S3–S6. Theoretical distribution of carotenoid isotopologs based on natural ^13^C abundance (~ 1.1%) is presented in Additional file 1: Fig. S8

Figure 5 collates mass spectra of all-*trans*-β-Car in the non-labeled and ^13^C-labeled samples analyzed by FTICR-MS and TQ-MS. Similar mass spectra, albeit with stronger backgrounds, were also obtained for the less abundant 9-*cis*-β-Car (Additional file 1: Fig.S10). The mass peaks of β-Car are clustered around *m*/*z* 536–537 in the non-labeled sample (Fig. 5a, b; Additional file 1: Fig.S10a, b). After the 7-d ^13^CO_2_ treatment, a second peak cluster comprising ^13^C-labeled isotopologs emerged at around *m*/*z* 575–576 (Fig. 5c, d; Additional file 1: Fig.S10c, d). The peaks of ^13^C-labeled isotopologs were much higher than the non-labeled ones, indicating that the majority of β-Car, both all-*trans* and 9-*cis*, were newly synthesized during the ^13^CO_2_ treatment.

We selected the mass peaks of all-*trans*-β-Car isotopologs in the TQ-MS data (Additional file 2: Tables S4, S6) as per the peak assignment of FTICR-MS in the non-labeled and ^13^C-labeled samples (Additional file 2: Tables S3, S5). Due to limited mass resolution, TQ-MS cannot distinguish mass peaks of [M]^+^ and [M + H]^+^ ions when they have similar *m*/*z* values following ^12^C/^13^C substitution (Δ mass = 1.003355) or protonation (Δ mass = 1.007276). FTICR-MS, at the resolving power used (100,000 at *m*/*z* 400), could separate most of the β-Car peaks in the ^13^C-labeled cluster, while it failed to resolve a few peaks in the non-labeled cluster (Additional file 2: Tables S3–S6). In case of overlap, peaks were regarded as the more abundant form. Thus, overlapping peaks of [M]^+^ and [M + H]^+^ were considered [M]^+^ in the TQ-MS analysis and [M + H]^+^ in the FTICR-MS analysis of β-Car. This led to a small overestimation of β-Car ΣDoL by TQ-MS because [M]^+^ has one more ^13^C atom compared to [M + H]^+^ at similar *m*/*z* (e.g. [^12^C_39_^13^C-M]^+^ and [^12^C_40_-M + H]^+^). Consequently, ΣDoL values of all-*trans*-β-Car were slightly higher when calculated from the TQ-MS data (ca. 1.4% and 86.1% for non-labeled and ^13^C-labeled sample, respectively) instead of the FTICR-MS data (ca. 1.2% and 84.2%) (Additional file 2: Tables S3–S6). Using the same peak assignment, we found comparable ΣDoL for 9-*cis*-β-Car shown in Additional file 1: Fig. S10: ca. 1.2% and 84.7% for non-labeled and ^13^C-labeled sample by TQ-MS, ca. 1.0% and 82.1% by FTICR-MS. Note that the peaks of all-*trans*- and 9-*cis*-β-Car were not completely separated in the ion chromatogram (Fig. 4g), which may partly explain the similar ΣDoL of these isomers.

Next, we estimated the size of NLP based on the relative abundance of non-labeled β-Car isotopologs in the ^13^C-labeled sample (i.e., the sum of BPI_*i*(norm)_ in the white cells of Additional file 2: Tables S5, S6). For calculation of NLP, it should not matter whether unresolved peaks of β-Car are considered [M]^+^ or [M + H]^+^ because the overlaps occurred exclusively within the non-labeled or the ^13^C-labeled peak cluster. Nevertheless, TQ-MS gave a somewhat smaller NLP of all-*trans*-β-Car (ca. 10.3%) than FTICR-MS did (ca. 12.2%) (Additional file 2: Tables S5, S6). The same also applied to 9-*cis*-β-Car (ca. 11.5% and 14.3% by TQ-MS and FTICR-MS, respectively). NLP is always 100% in non-labeled control.

**Fig. 6:**
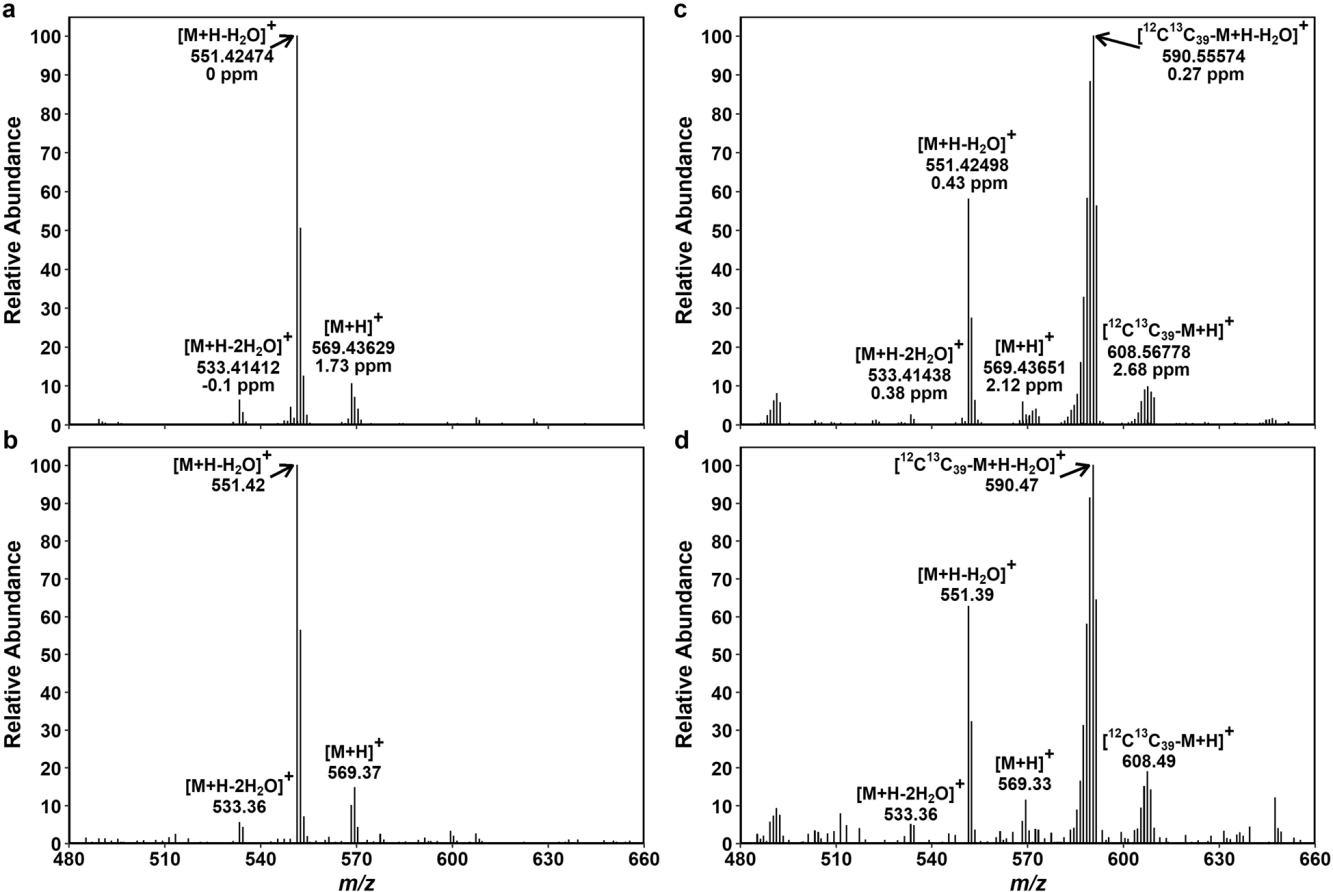
Mass spectra of Lut extracted from non-labeled and ^13^C-labeled Arabidopsis plants. FTICR-MS showing four different types of quasi-molecular ions of Lut, [M + H–2H_2_O]^+^, [M + H–H_2_O]^+^, [M]^+^ and [M + H]^+^, in a non-labeled (**a**) and a ^13^C-labeled (**c**) sample. Small peaks of ^13^C-labeled [M + H–2H_2_O]^+^ ion were detected in the same *m*/*z* region as non-labelled [M]^+^ and [M + H]^+^ ions in **c**. Deviations from the expected mass (Δ) are given in ppm. TQ-MS showing four types of quasi-molecular ions, [M + H–2H_2_O]^+^, [M + H–H_2_O]^+^, [M]^+^ and [M + H]^+^, in the same non-labeled (**b**) and ^13^C-labeled (**d**) samples as in **a** and **c**. Since TQ-MS cannot separate overlapping peaks of non-labeled [M]^+^ and [M + H]^+^ at *m*/*z* 569–571 and ^13^C-labeled [M]^+^ and [M + H]^+^ at *m*/*z* 607–608, they are regarded as [M + H]^+^. Peak assignment of these data is summarized in Additional file 2: Tables S7–S10

We followed the same procedure to assign the mass peaks of Lut. As already mentioned, [M + H–H_2_O]^+^ is the predominant ion of Lut (Fig. 6) [14, 18, 19]. FTICR-MS separated all mass peaks of Lut detected in non-labeled and ^13^C-labeled samples (Additional file 2: Tables S7, S9), while TQ-MS showed overlaps between [M]^+^ and [M + H]^+^ in both non-labeled and ^13^C-labeled *m*/*z* regions (Additional file 2: Tables S8, S10). Since the overlapping peaks in the TQ-MS data were considered [M + H]^+^, ΣDoL of Lut was slightly underestimated by TQ-MS in both non-labeled and ^13^C-labeled samples (ca. 1.0% and 74.1%, respectively) compared to FTICR-MS (ca. 1.3% and 75.0%) (Additional file 2: Tables S7–S10). Conversely, NLP was marginally overestimated by TQ-MS in the labeled sample (ca. 21.9% vs 21.0% by FTICR-MS).

**Fig. 7:**
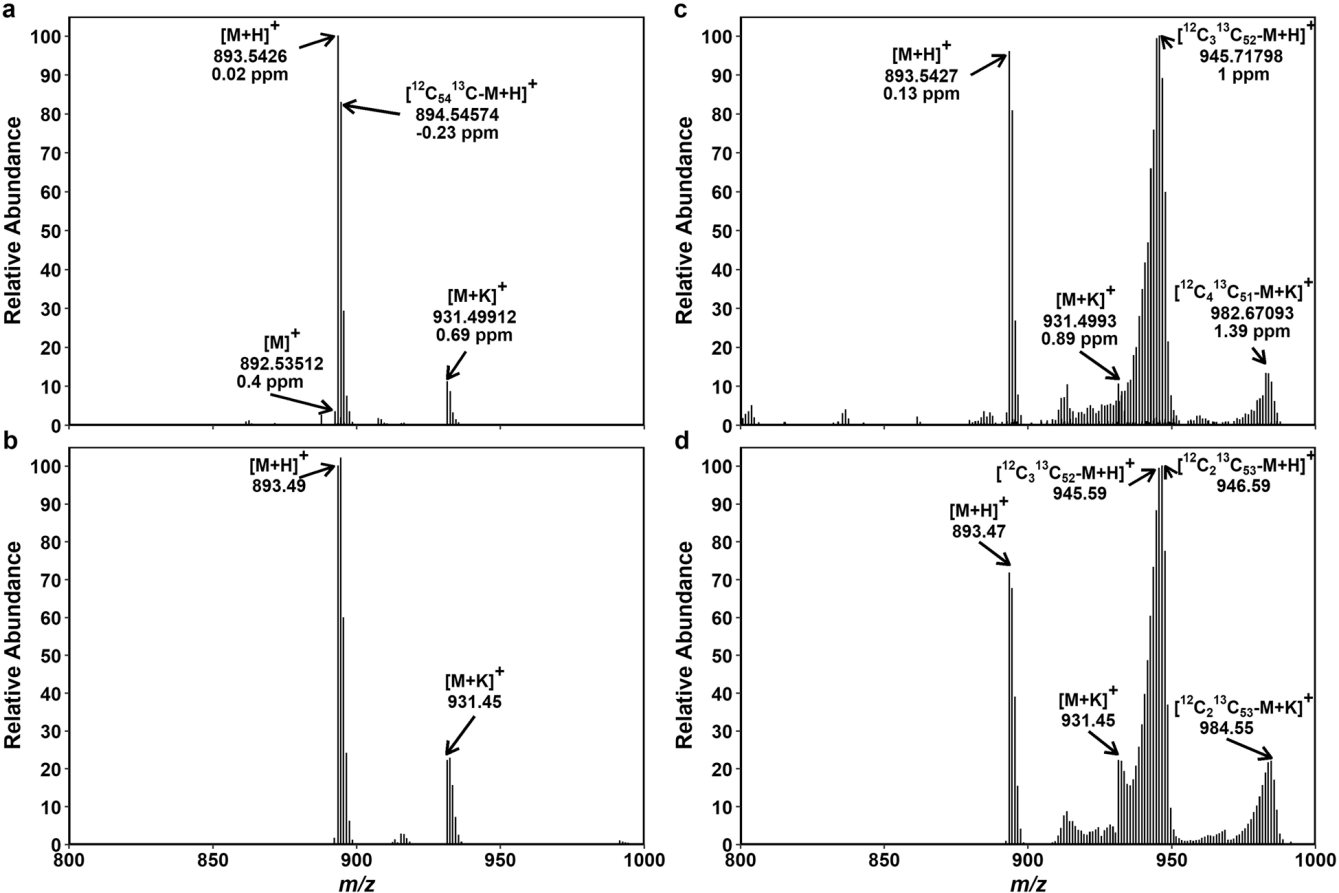
Mass spectra of Chl *a* extracted from non-labeled and ^13^C-labeled Arabidopsis plants. FTICR-MS showing three types of quasi-molecular ions of Chl *a*, [M]^+^, [M + H]^+^ and [M + K]^+^, in a non-labeled (**a**) and a ^13^C-labeled (**c**) sample. Deviations from the expected mass (Δ) are given in ppm. TQ-MS showing two types of quasi-molecular ions, [M + H]^+^ and [M + K]^+^, in the same non-labeled (**b**) and ^13^C-labeled (**d**) samples as in **a** and **c**. The [M]^+^ peak was hardly detected and thus not considered in the analysis of TQ-MS data. Mass peaks of ^13^C-labeled [M + H]^+^ and non-labeled [M + K]^+^ were overlapping at *m*/*z* 931–933 in **d**. The contribution of non-labeled [M + K]^+^ in this *m*/*z* region was estimated from the intensity of non-labeled [M + H]^+^ peaks and the ratio between [M + H]^+^ and [M + K]^+^ peaks found in **b** (1:0.26). For Chl, natural abundance of Mg isotopes (^24^Mg 79%, ^25^Mg 10% and ^26^Mg 11%) was taken into account to calculate their contributions to each mass peak. The estimated peak intensity of ^24^Mg-Chl as [M + H]^+^ was then considered representative of Chl *a* in the analysis of TQ-MS data. Peak assignment of these data is summarized in Additional file 2: Tables S11–S14

**Fig. 8:**
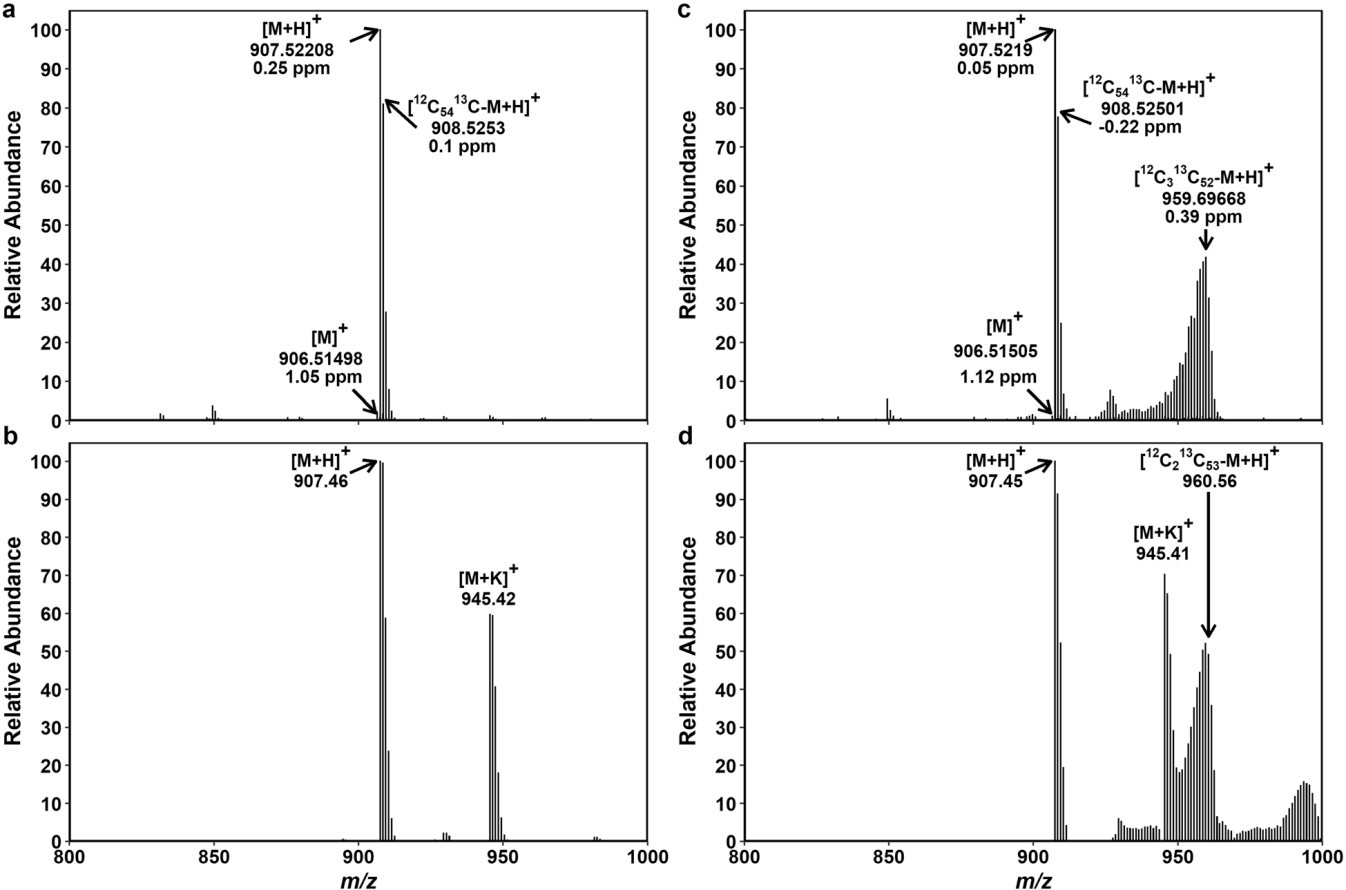
Mass spectra of Chl *b* extracted from non-labeled and ^13^C-labeled Arabidopsis leaves. FTICR-MS showing two types of quasi-molecular ions of Chl *b*, [M]^+^ and [M + H]^+^, in a non-labeled (**a**) and a ^13^C-labeled (**c**) sample. Deviations from the expected mass (Δ) are given in ppm. TQ-MS showing two types of quasi-molecular ions, [M + H]^+^ and [M + K]^+^, in the same non-labeled (**b**) and ^13^C-labeled (**d**) samples as in **a** and **c**. The [M]^+^ peak was hardly detected and thus not considered in the analysis of TQ-MS data. Mass peaks of ^13^C-labeled [M + H]^+^ and non-labeled [M + K]^+^ were overlapping at *m*/*z* 945–948 in **d**. The contribution of non-labeled [M + K]^+^ in this *m*/*z* region was estimated from the intensity of non-labeled [M + H]^+^ peaks and the ratio between [M + H]^+^ and [M + K]^+^ peaks found in **b** (1:0.74). For Chl, natural abundance of Mg isotopes (^24^Mg 79%, ^25^Mg 10% and ^26^Mg 11%) was taken into account to calculate their contributions to each mass peak. The estimated mass peak intensity of ^24^Mg-Chl as [M + H]^+^ was then considered representative of Chl *b* in the analysis of TQ-MS data. Peak assignment of these data is summarized in Additional file 2: Tables S15–S18

Unlike carotenoids, which are made solely of C and H (carotenes) or C, H and O (xanthophylls), Chls also contain N and Mg. While natural abundance of ^2^H (0.02%), ^17^O (0.04%), ^18^O (0.2%) and ^15^N (0.4%) are all low, two stable isotopes of Mg (^25^Mg 10%, ^26^Mg 11%) exist in significant amounts in nature alongside the most abundant ^24^Mg (79%). Peak assignment and analysis of Chl mass spectra must take into account Mg isotopes (^24^Mg/^25^Mg Δ mass = 1.000795; ^24^Mg/^26^Mg Δ mass = 1.997551) in addition to ^12^C/^13^C substitution and protonation. Furthermore, the number of possible formulae increases with increasing molecular mass. As a result, Chl mass spectra were difficult to analyze even by FTICR-MS. Moreover, formation of [M + K]^+^ adduct was seen in both FTICR-MS and TQ-MS data of Chl *a* (Fig. 7), and TQ-MS data of Chl *b* (Fig. 8b, d). Some TQ-MS data also showed a trace of [M + Na]^+^ in ^13^C-labeled samples. To simplify the analysis of Chl mass spectra, we focused on the [M + H]^+^ ion of ^24^Mg-Chl because ^13^C labeling of Chl should be independent of the type of ion produced by MS instruments and of the Mg isotope inserted in the protoporphyrin IX during Chl biosynthesis. The relative contributions of ^24^Mg-Chl, ^25^Mg-Chl and ^26^Mg-Chl to overlapping mass peaks were calculated based on the natural abundance of these Mg isotopes. As the peaks of non-labeled [M + K]^+^ and ^13^C-labeled [M + H]^+^ were partly overlapping (Figs. 7c, d and 8d), we estimated the intensity of non-labeled [M + K]^+^ peaks from the intensity of non-labeled [M + H]^+^ peaks in the same data, assuming the relative abundance of [M + K]^+^ and [M + H]^+^ peaks found in the non-labeled control (Figs. 7a, b and 8b). The intensity of non-labeled [M + K]^+^ was then subtracted from the overlapping peaks to obtain the intensity of ^13^C-labeled [M + H]^+^.

The peak assignment and calculation of Chl *a* and Chl *b* are summarized in Additional file 2: Tables S11–S18. For comparison, calculation is reported for both [M + H]^+^ and [M + K]^+^ ions in the non-labeled control (Additional file 2: Tables S11, S12, S16). As expected, ΣDoL did not substantially differ between the two ionization products ([M + H]^+^ and [M + K]^+^). Between the two instruments, TQ-MS gave higher ΣDoL for both non-labeled and ^13^C-labeled samples compared to FTICR-MS; the values obtained in the labeled sample were ca. 67.3% and 70.4% for Chl *a* (by FTICR-MS and TQ-MS; Additional file 2: Tables S13, S14) and ca. 54.9% and 57.5% for Chl *b* (Additional file 2: Tables S17, S18). The TQ-MS data underestimated NLP of Chl *a* in the ^13^C-labeled sample (ca. 18.8% and 20.7% by TQ-MS and FTICR-MS), whereas their NLP values were similar for Chl *b* (ca. 33.6% and 33.0%).

Overall, ΣDoL and NLP were largely comparable between the TQ-MS and FTICR-MS data, with deviations ranging between less than one and a few points after the 7-d ^13^CO_2_ labeling. Also the technical reproducibility was high for both parameters (Additional file 2: Table S2).

### Variations in ΣDoL and NLP among the 15 plants in the ^13^CO_2_-labeling experiment

**Fig. 9:**
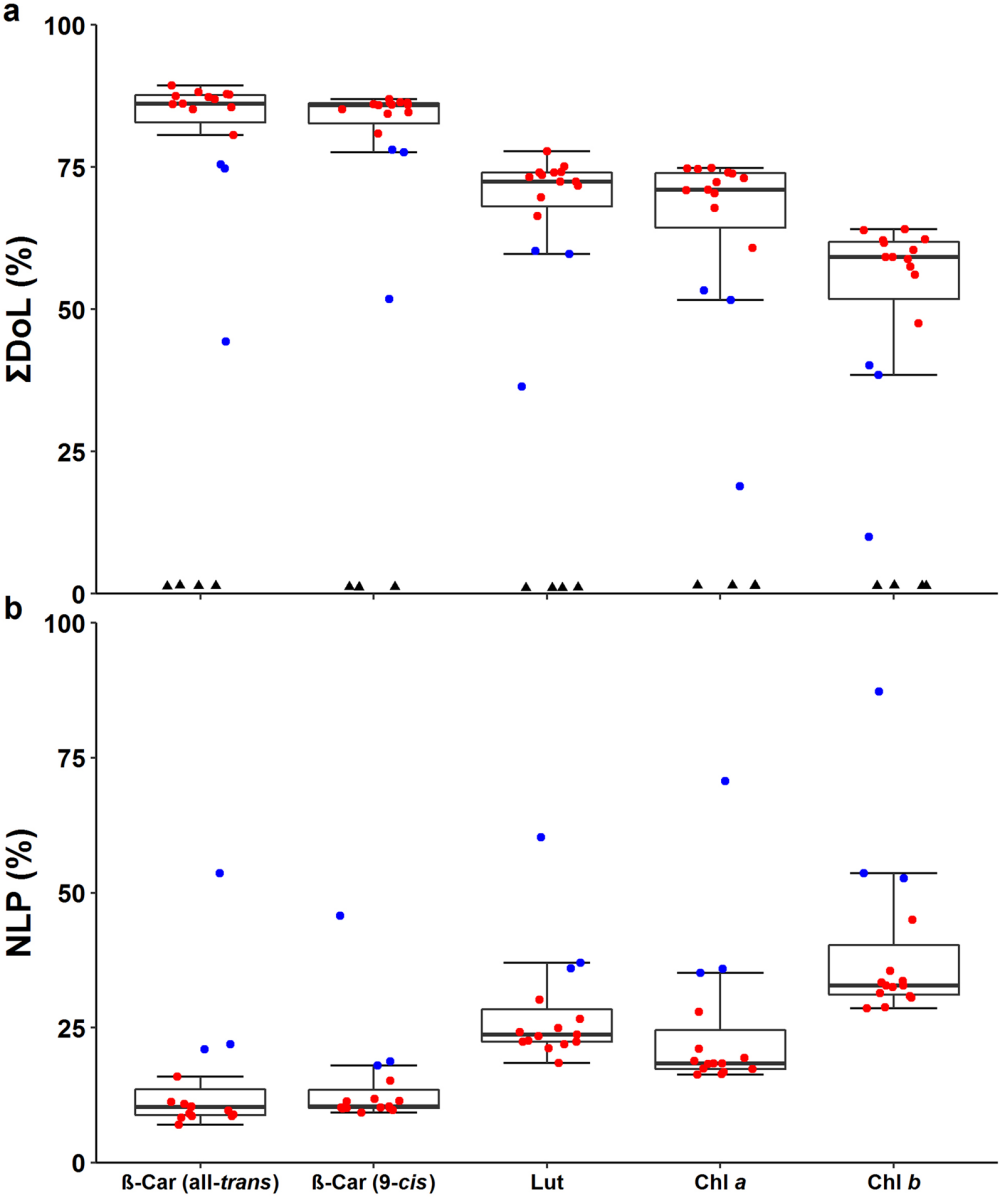
Labeled and non-labeled pigments in ^13^C-labeled Arabidopsis leaves harvested after 7-d ^13^CO_2_ labeling. **a** Degree of ^13^C labeling (ΣDoL) and **b** non-labeled pigment population (NLP) of all-*trans*- and 9-*cis*-β-Car, Lut, Chl *a* and Chl *b*. Red and blue symbols represent plants that had higher (*n* = 12) or lower (*n* = 3) ^13^C incorporation in pigments, respectively. Black triangles in **a** are control plants (*n* = 4) that stayed in the ambient air outside the labeling chamber. Data of the control plants are not shown in **b** since they all had 100% NLP. The box plots are based on the data of the ^13^C-labeled samples (i.e., red and blue symbols); the control plants (black triangles) shown in **a** are not included in the box plots. The thick horizontal line inside the box shows the median. The middle 50% of the data fall between the upper and lower end of the box. Data beyond the whisker boundaries are outliers

We analyzed ΣDoL and NLP in all samples using TQ-MS. Figure 9a shows box plots of ΣDoL. Three plants had lower ^13^C enrichment in the pigments; these were P6, P13 and P15 that displayed anthocyanin accumulation and reduced growth (Additional file 1: Fig. S3b). The smallest plant (P13) had the lowest ΣDoL for all pigments. Apart from these three, the other ^13^C-labeled plants were similar in terms of ΣDoL (Fig. 9a). β-Car had the highest average ΣDoL (86.5% ± 2.2 SD for all-*trans*; 85.4% ± 1.6 for 9-*cis*) and Chl *b* the lowest (59.4% ± 4.5). The ΣDoL was comparable for Lut (72.9% ± 2.8) and Chl *a* (71.5% ± 4.0). Notably, P13 showed substantially lower ΣDoL for Chl *a* (ca. 18.9%) than for Lut (ca. 36.4%). Far below the values of the ^13^C-labeled plants, ΣDoL of the control plants ranged between 1.0% and 1.5% for all pigments, as is expected from the 1.1% natural abundance of ^13^CO_2_ in the atmosphere.

NLP (Fig. 9b) shows a mirror image of ΣDoL (Fig. 9a). As seen for ΣDoL, the NLP values were similar in all ^13^C-labeled plants but the three (P6, P13 and P15). After the 7-d labeling, the average NLP of the 12 plants was no more than 9.9% ± 2.2 for all-*trans*-β-Car and 10.8% ± 1.6 for 9-*cis*-β-Car (Fig. 9b). These values are about a half of Chl *a* (18.9% ± 3.2) and Lut (23.5% ± 2.9) or 1/3 of Chl *b* (33.0% ± 4.3). The smallest plant (P13) had the largest NLP for all pigments, showing again a large difference between Chl *a* (ca. 70.7%) and Lut (ca. 60.3%).

**Fig. 10:**
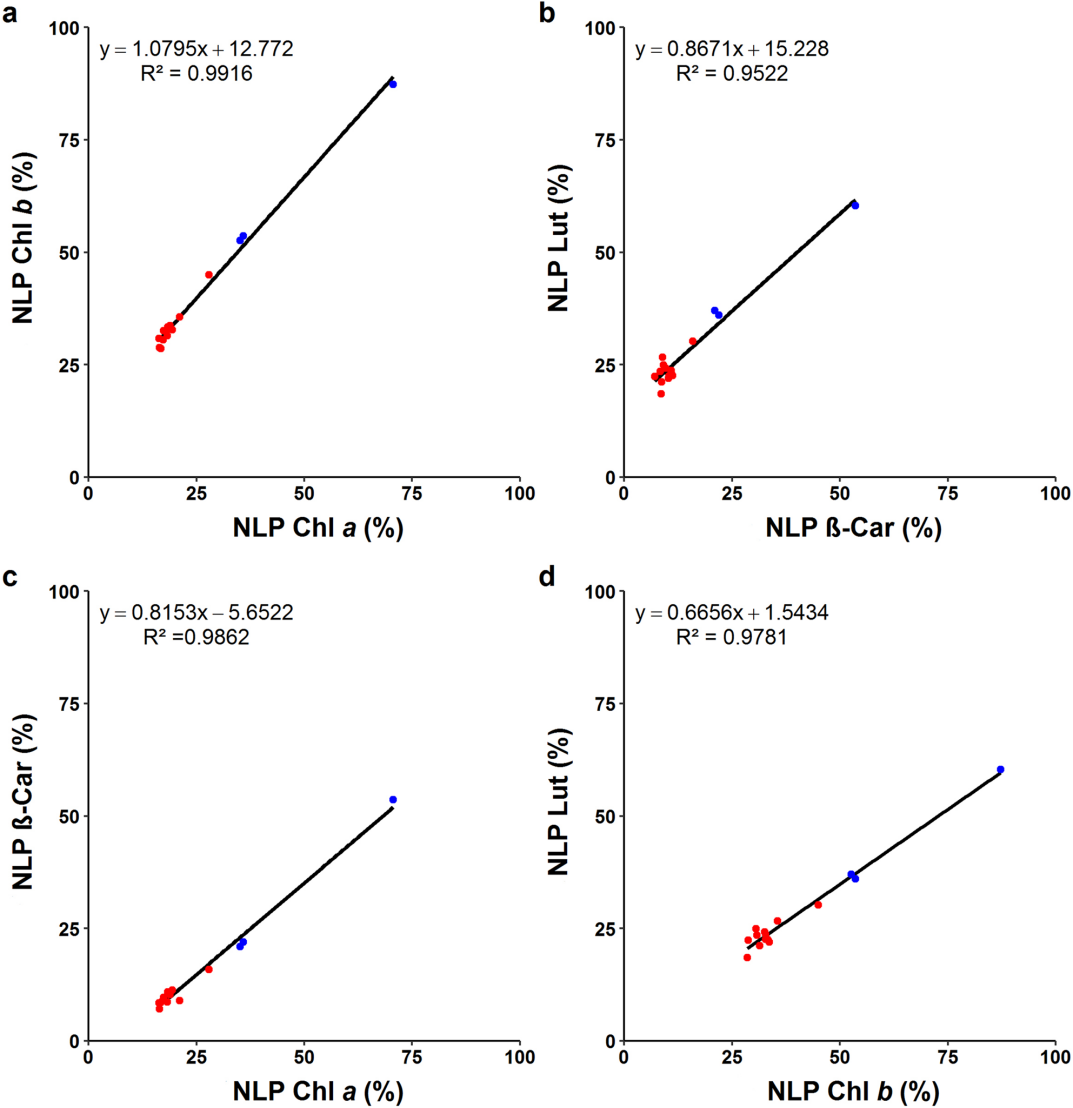
Correlation between non-labeled population (NLP) of pigments extracted from Arabidopsis leaves after 7-d ^13^CO_2_ labeling. **a** Chl *a* and Chl *b*. **b** All-*trans*-β-Car and Lut. **c** Chl *a* and all-*trans*-β-Car. **d** Chl *b* and Lut. Red and blue symbols represent plants that had higher (*n* = 12) or lower (*n* = 3) ^13^C incorporation in pigments, respectively

Having seen the non-uniform ^13^C enrichment patterns in different pigments of the ^13^C-labeled plants (Fig. 9), we made pairwise comparisons of NLP between the pigments (Fig. 10). All four comparisons revealed a positive linear correlation but the slope of regression lines differed. The slope was roughly one in the comparison between the two Chls, although NLP was always smaller for Chl *a* than for Chl *b* (Fig. 10a). The correlation between the two carotenoids had a slope of less than 0.9 (Fig. 10b), suggesting a smaller variation in Lut per unit change in all-*trans*-β-Car. The Chl-carotenoid comparisons, i.e., Chl *a vs* all-*trans*-β-Car (Fig. 10c; slope ~ 0.8) and Chl *b vs* Lut (Fig. 10d; slope < 0.7), indicated larger variations for Chls than for carotenoids among the 15 plants. Yet, NLP was consistently smaller for carotenoids than for Chls. The same comparisons made for ΣDoL (Additional file 1: Fig. S11) displayed the same trends in the opposite direction.

The large variations in NLP and ΣDoL found between the non-labeled and ^13^C-labeled plants as well as between the non-stressed and stressed plants (Figs. 9, 10; Additional file 1: Fig. S11) are in marked contrast to the similarity in their pigment composition (Fig. 3).

## Discussion

### Chamber for long-term ^13^CO_2_ labeling

^13^CO_2_ labeling offers a means to trace the fate of carbon assimilated by photoautotrophic organisms. After fixation into sugars, further metabolization can be detected by GC–MS, LC–MS or NMR [20–22] to study the networks of carbon metabolism in plant tissues. The interest in metabolic flux analysis has been growing in plant research, despite the challenges that are inherent to multi-compartment cells and multicellular samples [23–26]. For instance, ^13^CO_2_ pulse-chase experiments have been conducted for flux analysis of central metabolism in intact leaves and whole Arabidopsis rosettes [27–31]. For short-term (seconds to minutes) labeling to capture rapid ^13^C incorporation and enrichment in primary metabolites, labeling chambers and leaf cuvettes must have a small volume to ensure minimal time lag after switching between ambient CO_2_ and ^13^CO_2_ [27–31]. Some chambers also allow instant quenching (flash-freezing) to preserve metabolic state of the sample. Small labeling chambers and cuvettes were also used to track ^13^C down the plastidic 2*C*-methyl-d-erythritol-4-phosphate (MEP) pathway and isoprene biosynthesis [32–36].

^13^CO_2_ labeling is an established approach in plant research to investigate phenomena that develop over hours and days or even weeks and months. Ecological and ecophysiological studies, such as investigation of above- and belowground carbon allocation in trees [37–40], employ δ^13^C measurements by isotope ratio mass spectrometer (IRMS) following in situ ^13^CO_2_ labeling. The δ^13^C method can also be employed for laboratory experiments to trace metabolization and translocation of ^13^C-labeled assimilates [41]. When combined with hydroponic cultivation, dual labeling with ^13^CO_2_ and ^15^N (in the form of ^15^NH_4_NO_3_, ^15^NH_4_^15^NO_3_ or K^15^NO_3_ in nutrient solution) enables simultaneous tracking of C and N allocation [41]. While ^15^N feeding was successfully applied to unveil leaf proteome turnover [2, 3], attempts are being made with ^13^CO_2_ to concomitantly analyze turnover of metabolites and proteins [42, 43]. Chambers for long-term ^13^CO_2_ labeling typically have a large volume to treat multiple plants in parallel [41–44]. Even an entire walk-in climate chamber can be used for ^13^CO_2_ labeling [45], budget permitting. In order to both monitor and control the conditions during the experiments, long-term labeling chambers are often equipped with environmental sensors and control devices [41, 42, 44].

The choice of chamber design depends on research goals and available resources. Our labeling chamber for long-term turnover analysis belongs to the latter type. It has a volume of ca. 70 L for simultaneous labeling of up to 15 small plants and is furnished with temperature, humidity, light and pressure sensors besides the IRGA for measuring [CO_2_] (Figs. 1, 2). Due to the low sensitivity of LI-840 for ^13^CO_2_, a preliminary experiment with normal CO_2_ was necessary to establish a labeling protocol (Additional file 1: Figs. S5, S6). Not having [^13^CO_2_] measurement is a major limitation of the current setup of the labeling chamber, which may result in variable [^13^CO_2_] between experiments. This limitation can be removed in the future by installing an IRGA or other instrument that can measure ^13^CO_2_. One could also consider the use of IRMS to check the similarity or dissimilarity of ^13^C assimilation by the plants in different experiments. If a ^13^CO_2_ gas analyzer is not available, as was the case in thepresent work, flow rate protocols developed in normal CO_2_ can be used to provide the experimental conditions in ^13^CO_2_ that are as close to the set values as possible. It is then imperative that very similar plants be used in both CO_2_ and ^13^CO_2_ experiments (Additional file 1: Fig. S2–S4). Also, the importance of homogenous conditions inside the chamber (Fig. 2a) cannot be stressed enough; environmental heterogeneity can have cumulative effects on ^13^CO_2_ labeling of plants (replicates) over a long period. In the labeling experiment, except for the visibly stressed P6, P13 and P15, the other plants were similar in terms of visual phenotype (Additional file 1: Fig. S3), pigment composition (Fig. 3) as well as NLP and ΣDoL (Fig. 9). Although [^13^CO_2_] was not measured in this study, our chamber and experimental protocols apparently provided adequate conditions for long-term ^13^CO_2_ labeling.

### NLP of carotenoids

Similar ΣDoL and NLP values of the non-stressed plants (Fig. 9) underscore high reproducibility (both technical and biological) of the methods. Whilst the number of unresolved mass peaks was greater in the TQ-MS data than in the FTICR-MS data (Additional file 2: Tables S3–S18), high pigment recovery and reproducibility of the TQ-MS system could be validated (Additional file 2: Tables S1, S2). Below, we discuss NLP of carotenoids (Lut and β-Car) and Chls (Chl *a* and Chl *b*) obtained by TQ-MS.

Previously we described a method of Lut isotopolog profiling in ^13^C-labeled leaf pigment extracts [14]. Using the method, NLP was calculated for Lut extracted from leaves of the 7-d ^13^C-labeled Arabidopsis plants (Fig. 9b). The data of the non-stressed plants are scattered around the median, which was much lower than the NLP in the stressed plants. The deviations between the TQ-MS-based and FTICR-MS-based NLP are < 1 point for Lut, despite the unresolved peaks in the TQ-MS data at *m*/*z* 569‒571 and *m*/*z* 607‒608 (Additional file 2: Tables S8, S10) [14]. This is because none of the overlapping peaks belongs to the predominant ion [M + H–H_2_O]^+^. The relative intensity (BPI_*i*(norm)_) of the overlapping peaks is too low to affect ΣDoL and NLP substantially. Moreover, the overlaps between [M]^+^ and [M + H]^+^ are of no consequence to NLP when they appear within non-labeled or labeled peak cluster; only the peak assignment of small overlaps between non-labeled and labeled isotopologs at *m*/*z* 569‒571 affects the calculation of NLP. The relative intensity in this *m*/*z* region is no more than 3% of the total, about a half of which is assigned to non-labeled [M]^+^ and [M + H]^+^ and the other half to ^13^C-labeled [M + H–2H_2_O]^+^ (Additional file 2: Table S9). When the abundance of the latter diminishes in less strongly labeled samples (e.g. in experiments with shorter labeling), between-cluster peak overlap will not be an issue for Lut.

Given that all ionization products must have the same ^13^C labeling pattern, an alternative way to analyze Lut data is to avoid overlapping peak regions altogether and use only [M + H–H_2_O]^+^, which constitutes > 80% of our Lut mass spectra (Fig. 6; Additional file 1: Fig. S9a–c). The NLP values thus calculated are ca. 20.9% and 20.6%, respectively, for the FTICR-MS and TQ-MS data shown in Additional file 2: Tables S9, S10, instead of ca. 21.0% and 21.9% considering all four ions. Regardless of whether the calculation includes all ions or only [M + H–H_2_O] ^+^, similar NLP values can be obtained for Lut, with very minor differences between FTICR-MS and TQ-MS. We note that peak assignment and calculation of DoL and NLP basically follow the same procedures for all xanthophylls that are typically found in chloroplasts.

New in the present study is the assignment of β-Car and Chl isotopolog peaks to enable turnover analysis of different pigments in the same sample. Two well-separated peak clusters characterize the mass spectra of all-*trans*-β-Car in ^13^C-labeled samples (Fig. 5c, d; Additional file 1: Fig. S9d–f): a labeled cluster at around *m*/*z* 575–576 and a non-labeled cluster at around *m*/*z* 536–537. The latter reflects the natural abundance of ^12^C/^13^C, as seen in the control (Fig. 5a, b) and predicted by the simulation (Additional file 1: Fig. S8). Since the unresolved mass peaks of β-Car appear solely within non-labeled or labeled cluster, they should not affect the calculation of NLP. Even so, TQ-MS slightly underestimated NLP of β-Car in the ^13^C-labeled sample (ca. –1.9 and –2.8 points for all-*trans*- and 9-*cis*-β-Car, respectively) compared to FTICR-MS. The discrepancy between the two instruments declines to ca. 1.1 and 1.7 points when NLP of the FTICR-MS data is calculated from the major [M + H]^+^ ion alone, rather than both [M]^+^ and [M + H]^+^ (Additional file 2: Table S5).

All-*trans*-β-Car is the predominant form of Car in PSII and PSI [11, 46]. While α-Car may also be found in leaf pigment extracts, especially (but not only) in shade-tolerant species [47, 48], the α-Car level is typically low in Arabidopsis leaves. Strong light and heat can trigger *cis*–*trans* isomerization in carotenoids [49, 50], which can be separated by C_30_ reversed-phase HPLC column [51, 52]. In the present study, we had moderate light and temperature conditions in the labeling chamber (Additional file 1: Fig. S7a, b) and pigments were extracted in chilled acetone under dim light. Still, 9-*cis*-β-Car and 9-*cis*-Neo were detected in all samples (Fig. 4a). Formation of these 9-*cis* isomers was specific, as neither 13-*cis* and 15-*cis* isomers of β-Car and Neo nor any *cis* isomers of Vio, Anthera and Lut were found. In fact, 9-*cis*-β-Car is a native constituent of cytochrome *b*_6_*f* complex [53–55]. Binding of 9-*cis*-Neo to PSII light-harvesting antenna complexes is also well-established [56–59]. Interestingly, even though the two β-Car isomers differ greatly in the concentration (Fig. 4a), their NLP values after the 7-d ^13^CO_2_ labeling were equally low (Fig. 9b).

### NLP of Chls

The ^13^C-labeled isotopolog peaks are more broadly distributed than non-labeled ones. This broadening is particularly manifest in the mass spectra of Chls (Figs. 7c, d and 8c, d) consisting of a porphyrin ring and a phytol side chain. At the center of porphyrin is Mg with three naturally abundant isotopes (^24^Mg, ^25^Mg and ^26^Mg), which, however, hardly broadens the mass spectra of non-labeled Chls (Figs. 7a, b and 8a, b). The broadening of Chl mass spectra is therefore solely ascribable to ^13^C enrichment. In accordance, similarly broad mass spectra were reported for ^13^C-labeled pheophytin following the removal of Mg from ^13^C-labeled Chl [60].

The peak cluster of ^13^C-labeled Chls extends over a wide *m*/*z* region, with the strongly labeled isotopologs (^13^C_55_ to ^12^C_15_^13^C_40_) accounting for a large part of ΣDoL (Additional file 2: Tables S13, S14, S17 and S18). The labeled [M + H]^+^ cluster is tailing off at lower *m*/*z*, as can be recognized in Figs. 7 and 8 despite the partial overlap with non-labeled [M + K]^+^ peaks. The mass peaks in this low *m*/*z* region have been attributed to isotopologs having ^13^C-labeld porphyrin with non-labeled phytol or ^13^C-labeled phytol with non-labeled porphyrin [60]. Our Chl mass spectra revealed a small sub-cluster at around ^12^C_35_^13^C_20_ (*m*/*z* ~ 913 for Chl *a* and ~ 927 for Chl *b;* Figs. 7 and 8), corresponding to non-labeled porphyrin with ^13^C-labeled phytol. The existence of this sub-cluster is most evident in the absence of [M + K]^+^ in the FTICR-MS data of Chl *b* (Fig. 8c). In contrast, mass peak distribution was continuous for ^13^C-labeled porphyrin with non-labeled phytol, suggesting variable ^13^C enrichment patterns of the porphyrin moiety synthesized from glutamate.

Glutamate is rather slowly labeled by ^13^CO_2_ and also slowly unlabeled during subsequent chase in ambient CO_2_ [28, 41, 42]. In comparison, glyceraldehyde-3-phosphate and pyruvate, the two precursors of plastidic isoprenoid biosynthesis via the MEP pathway leading to carotenoids and phytol, are rapidly labeled by ^13^CO_2_ in illuminated leaves [28]. The Chl molecules comprising labeled and non-labeled moieties are thought to arise from recycling of de-esterified chlorophyllide and phytol [60]. By using radioactive ^3^H-labeling, incorporation of phytol in Chl and tocopherol has been demonstrated in Arabidopsis seedlings [61]. It should be noted, however, that Chl molecules, which are “newly” synthesized from recycled chlorophyllide and recycled phytol, will be indistinguishable from preexisting “old” molecules based on their mass. Should such complete recycling occur, it would result in overestimation of NLP. How often Chl molecules are recycled in leaves is unknown. According to our data from the 7-d labeled Arabidopsis leaves, the relative abundance of isotopologs with labeled and non-labeled moieties was 5–9% of the total Chl pool (Figs. 7 and 8; Additional file 2: Tables S13, S14, S17 and S18). Similar values (< 10%) were also found for pheophytin prepared from Chl *a* of *Synechocystis* sp. PCC 6803 after 2-d labeling with ^13^C-glucose and H^13^CO_3_^−^ [60].

We were unable to resolve all peaks in the pigment mass spectra, in particular for Chls (Additional file 2: Tables S11–S18). Higher resolving power than was used in this study would be desirable for better peak assignment. The analysis of Chl mass spectra can be simplified through Mg removal by weak acid treatment [60], although this will require separate sample preparation and analysis for Chls and carotenoids. Formation of alkali metal adducts is a common phenomenon for some compounds in positive ion mode. We estimated the contribution of [M + K]^+^ in the overlapping peak region of ^13^C-labeled Chl mass spectra (Figs. 7c, d and Fig. 8d) based on the [M + K]^+^:[M + H]^+^ ratio found in the control (Figs. 7a, b and Fig. 8b). This ratio was highly reproducible in the mass spectra of the four control plants (± 6–8% SD for Chl *a*, ± 4% SD for Chl *b*). We assumed that the ^13^C-labeled plants had similar levels of K (hence also similar [M + K]^+^:[M + H]^+^ ratios) in leaves. As K contents may vary in different tissues, genotypes and species under different conditions, careful choice of control is essential to estimate the relative intensity of [M + K]^+^ peaks in this way. Additionally, the choice of ionization technique can partly ameliorate the problem with alkali metal adducts [62]; in the present study, APCI (in FTICR-MS) induced less alkali metal adduction than ESI did (in TQ-MS) (Figs. 7 and 8).

The calculation based on [M + H]^+^ of ^24^Mg-Chl indicated ca. 18.8% and 20.7% NLP for Chl *a* (by TQ-MS and FTICR-MS, respectively) and ca. 33.6% and 33.0% for Chl *b* (Additional file 2: Tables S13, S14, S17 and S18). The discrepancy between the two instruments is somewhat larger for Chl *a* (ca. 2.2 points) and β-Car (ca. 1.1 and 1.7 points for all-*trans* and 9-*cis*) than for Chl *b* and Lut (< 1 point). In view of the variations among the non-stressed replicate plants shown in Fig. 9b, this level of under- or overestimation is within a tolerable range.

### Different NLP of carotenoids and Chls

Cells synthesize new molecules as they grow. Growth-driven incorporation of ^13^C strongly dilutes NLP in long-term labeling experiments. After the 7-d ^13^CO_2_ labeling, all pigments had larger NLP in the stressed plants (Fig. 9b) showing reduced growth (Additional file 1: Fig. S3b). If growth is the only process that dilutes NLP and there is no change in pigment concentration, the entire set of pigments is expected to show more or less the same decline in NLP. Yet, our analysis revealed distinct NLP for carotenoids and Chls (Fig. 9b), suggesting different turnover of these pigments.

Our previous studies using radioactive ^14^CO_2_ labeling have shown the turnover of Chl *a* and all-*trans*-β-Car in mature leaves of Arabidopsis in the light [12, 13]. Both pigments had ^14^C incorporation already after 30-min pulse labeling, whereas Chl *b* and xanthophylls did not. The rapid labeling of Chl *a* and all-*trans*-β-Car without changes in their concentration is indicative of high turnover, presumably in connection with the D1 damage and repair [12, 13]. The D1 protein of PSII reaction center is known to undergo high turnover in illuminated leaves [6–8]. Some of the Chl *a* and all-*trans*-β-Car molecules, which are bound in the reaction center and core complex of PSII [11], seem to be degraded and replaced by newly synthesized molecules during the repair cycle. Between the two, all-*trans*-β-Car had a smaller NLP after the 7-d ^13^CO_2_ labeling (Figs. 9b, 10c). This may be explained by different localization of these pigments; while all-*trans*-β-Car is mostly bound to the core complexes of PSII and PSI [11, 46], Chl *a* is universally found in all photosynthetic pigment-protein complexes. The proportion of molecules undergoing turnover in PSII is thus larger for all-*trans*-β-Car than for Chl *a*. Moreover, NLP of Chl *a* may be underestimated due to complete recycling of chlorophyllide and phytol, should this happen. Analogously, lower NLP of Lut compared to Chl *b* (Figs. 9b, 10d), the two pigments that co-localize in light-harvesting antenna complexes [56–59, 63], may be a sign of Chl recycling, although higher turnover of Lut cannot be ruled out. If 9-*cis*-β-Car is specifically bound to cytochrome *b*_6_*f* complex [53–55], the similar NLP values found for all-*trans*- and 9-*cis*-β-Car (Fig. 9b) may imply medium turnover of 9-*cis*-β-Car in cytochrome *b*_6_*f*, i.e., not as high as all-*trans*-β-Car in PSII but higher than that in PSI. Yet, given the incomplete chromatographic separation of all-*trans*- and 9-*cis*-β-Car (Fig. 4g), their mass spectra (Fig. 5; Additional file 1: Fig. S10) may contain some signals from each other. Better separation is needed to estimate NLP of the less abundant 9-*cis*-β-Car (Fig. 4a).

## Conclusions

With these combined methods for long-term ^13^CO_2_ labeling and parallel determination of pigment concentration and NLP established, it is now possible to study photosynthetic pigment turnover. While we did not do time course analysis, the results presented in Figs. 9 and 10 point to distinct turnover rates of carotenoids and Chls. Future experiments, including the xanthophyll-cycle pigments that were left out of the scope of this study, could throw light on active maintenance and adjustment of photosynthetic pigments in leaves. Since changes in ^13^C enrichment can be analyzed in a wide range of compounds using appropriate MS methods, long-term ^13^CO_2_ labeling chamber, like the one described above, can facilitate investigations of dynamic turnover of various metabolites and macromolecules in plants on a time scale of hours to days.

## Supplementary Information

**Additional file 1:** **Fig. S1.** Cultivation of Arabidopsis plants in plastic cups. **Fig. S2.** Rosette leaves of Arabidopsis plants before and after 7-d exposure to the different airs (day 8). **Fig. S3**. 15 Arabidopsis plants before and after 7-d exposure to ^13^CO_2_ in the labeling chamber. **Fig. S4**. 15 Arabidopsis plants before and after 7-d exposure to normal CO_2_ in the labeling chamber. **Fig. S5**. Flow rates of CO_2_-free air and CO_2_ as well as real-time readings of the CO_2_ concentration and overpressure inside the labeling chamber. **Fig. S6**. CO_2_ concentration measured by LI-840. **Fig. S7**. Conditions inside the ^13^CO_2_ labeling chamber. **Fig. S8**. Simulation of carotenoid isotopolog distribution based on the natural ^13^C abundance. **Fig. S9**. Mass spectra of Lut, all-*trans*-β-Car and Chl *a* from the spike tests of TQ-MS. **Fig. S10**. Mass spectra of 9-*cis*-β-Car extracted from non-labeled and ^13^C-labeled Arabidopsis plants.  **Fig. S11**. Correlation between ΣDoL of different pigments extracted from Arabidopsis leaves after 7-d ^13^CO_2_ labeling.

**Additional file 2: Table S1. **Pigment recovery of all-*trans*-β-Car, Lut and Chl *a.* **Table S2. **Reproducibility of ΣDoL and NLP calculated from TQ-MS data. **Table S3**. Peak assignment and calculation of BPI*i*, BPI*i*(norm) and DoL*i* for individual isotopologs of all-*trans*-β-Car from a non-labeled Arabidopsis plant obtained by FTICR-MS. **Table S4**. Peak assignment and calculation of BPI*i*, BPI*i*(norm) and DoL*i* for individual isotopologs of all-*trans*-β-Car from a non-labeled Arabidopsis plant obtained by TQ-MS. **Table S5**. Peak assignment and calculation of BPI*i*, BPI*i*(norm) and DoL*i *for individual isotopologs of all-*trans*-β-Car from a ^13^C-labeled Arabidopsis plant obtained by FTICR-MS. **Table S6**. Peak assignment and calculation of BPI*i*, BPI*i*(norm) and DoL*i *for individual isotopologs of all-*trans*-β-Car from a ^13^C-labeled Arabidopsis plant obtained by TQ-MS. **Table S7**. Peak assignment and calculation of BPI*i*, BPI*i*(norm) and DoL*i *for individual isotopologs of Lut from a non-labeled Arabidopsis plant obtained by FTICR-MS. **Table S8**. Peak assignment and calculation of BPI*i*, BPI*i*(norm) and DoL*i *for individual isotopologs of Lut from a non-labeled Arabidopsis plant obtained by TQ-MS. **Table S9**. Peak assignment and calculation of BPI*i*, BPI*i*(norm) and DoL*i *for individual isotopologs of Lut from a ^13^C-labeled Arabidopsis plant obtained by FTICR-MS. **Table S10**. Peak assignment and calculation of BPI*i*, BPI*i*(norm) and DoL*i *for individual isotopologs of Lut from a ^13^C-labeled Arabidopsis plant obtained by TQ-MS. **Table S11**. Peak assignment and calculation of BPI*i*, BPI*i*(norm) and DoL*i *for individual isotopologs of Chl *a *from a non-labeled Arabidopsis plant obtained by FTICR-MS. **Table S12**. Peak assignment and calculation of BPI*i*, BPI*i*(norm) and DoL*i *for individual isotopologs of Chl *a *from a non-labeled Arabidopsis plant obtained by TQ-MS. **Table S13**. Peak assignment and calculation of BPI*i*, BPI*i*(norm) and DoL*i *for individual isotopologs of Chl *a *from a ^13^C-labeled Arabidopsis plant obtained by FTICR-MS. **Table S14**. Peak assignment and calculation of BPI*i*, BPI*i*(norm) and DoL*i *for individual isotopologs of Chl *a *from a ^13^C-labeled Arabidopsis plant obtained by TQ-MS. **Table S15**. Peak assignment and calculation of BPI*i*, BPI*i*(norm) and DoL*i *for individual isotopologs of Chl *b *from a non-labeled Arabidopsis plant obtained by FTICR-MS. **Table S16**. Peak assignment and calculation of BPI*i*, BPI*i*(norm) and DoL*i *for individual isotopologs of Chl *b *from a non-labeled Arabidopsis plant obtained by TQ-MS. **Table S17**. Peak assignment and calculation of BPI*i*, BPI*i*(norm) and DoL*i *for individual isotopologs of Chl *b *from a ^13^C-labeled Arabidopsis plant obtained by FTICR-MS. **Table S18**. Peak assignment and calculation of BPI*i*, BPI*i*(norm) and DoL*i *for individual isotopologs of Chl *b *from a ^13^C-labeled Arabidopsis plant obtained by TQ-MS.

## Data Availability

The datasets obtained and/or analyzed during the current study are available from the corresponding author on request.
