# Supplementary material for: Combination of long-term 13CO2 labeling and isotopolog profiling allows turnover analysis of photosynthetic pigments in Arabidopsis leaves
Source: Plant Methods. 2022 Oct 1;18:114. doi: 10.1186/s13007-022-00946-3 (PMC9526918; doi:10.1186/s13007-022-00946-3)
Supplement: Supplementary file 1 — Additional file 1: Fig. S1. Cultivation of Arabidopsis plants in plastic cups. Fig. S2. Rosette leaves of Arabidopsis plants before and after 7-d exposure to the different airs (day 8). Fig. S3. 15 Arabidopsis plants before and after 7-d exposure to 13CO2 in the labeling chamber. Fig. S4. 15 Arabidopsis plants before and after 7-d exposure to normal CO2 in the labeling chamber. Fig. S5. Flow rates of CO2-free air and CO2 as well as real-time readings of the CO2 concentration and overpressure inside the labeling chamber. Fig. S6. CO2 concentration measured by LI-840. Fig. S7. Conditions inside the 13CO2 labeling chamber. Fig. S8. Simulation of carotenoid isotopolog distribution based on the natural 13C abundance. Fig. S9. Mass spectra of Lut, all-trans-β-Car and Chl a from the spike tests of TQ-MS. Fig. S10. Mass spectra of 9-cis-β-Car extracted from non-labeled and 13C-labeled Arabidopsis plants. Fig. S11. Correlation between ΣDoL of different pigments extracted from Arabidopsis leaves after 7-d 13CO2 labeling. [file 13007_2022_946_MOESM1_ESM.pdf]

Additional file 1

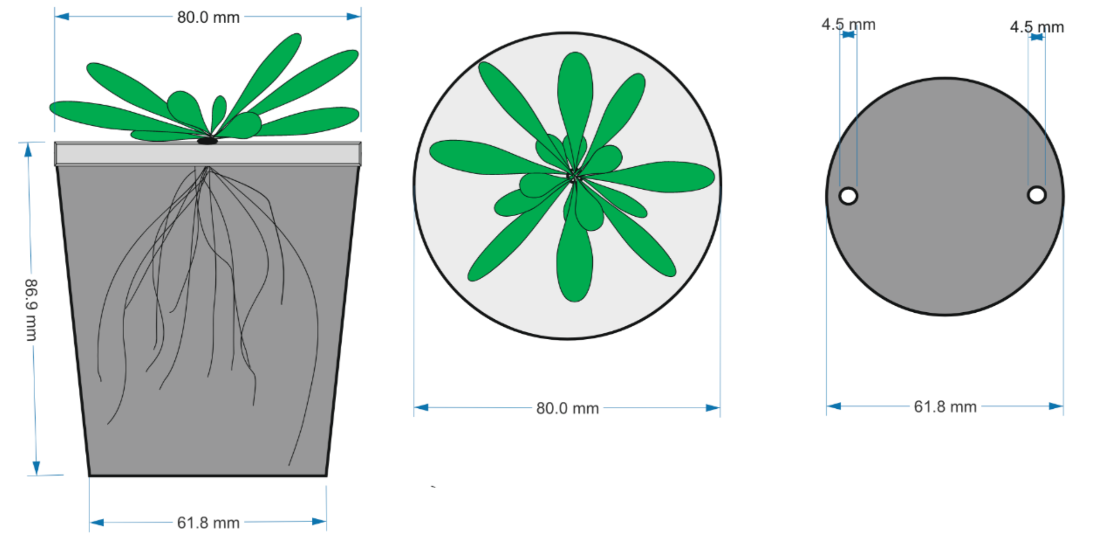

Fig. A1. Cultivation of Arabidopsis plants in plastic cups. Leaves can grow out of the cup (300 mL volume) through a hole (3 mm diameter) made in the center of the plastic lid. The lid separates the rosette from the root system growing in moist soil inside the cup, thus minimizing the impact of root and soil respiration on the CO_2 composition inside the labeling chamber. The lid is covered with aluminum foil to suppress growth of green algae on the soil surface. Two holes (4.5 mm diameter) are made in the bottom of the cup for bottom watering.

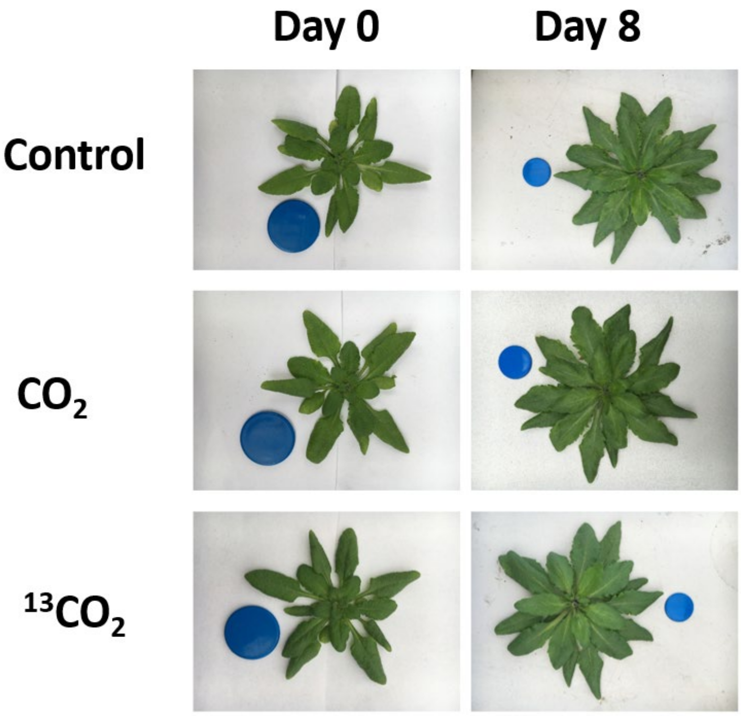

Fig. A2. Rosette leaves of Arabidopsis plants before (day 0) and after 7-d exposure to the different airs (day 8). All plants were grown in the same conditions prior to the experiment. Control: Plants stayed in the ambient air outside the labeling chamber. CO_2 and $^{13}CO_2$: Plants were exposed to CO_2 or $^{13}CO_2$ in the labeling chamber. Blue reference chips next to the plants have a diameter of 2 cm.

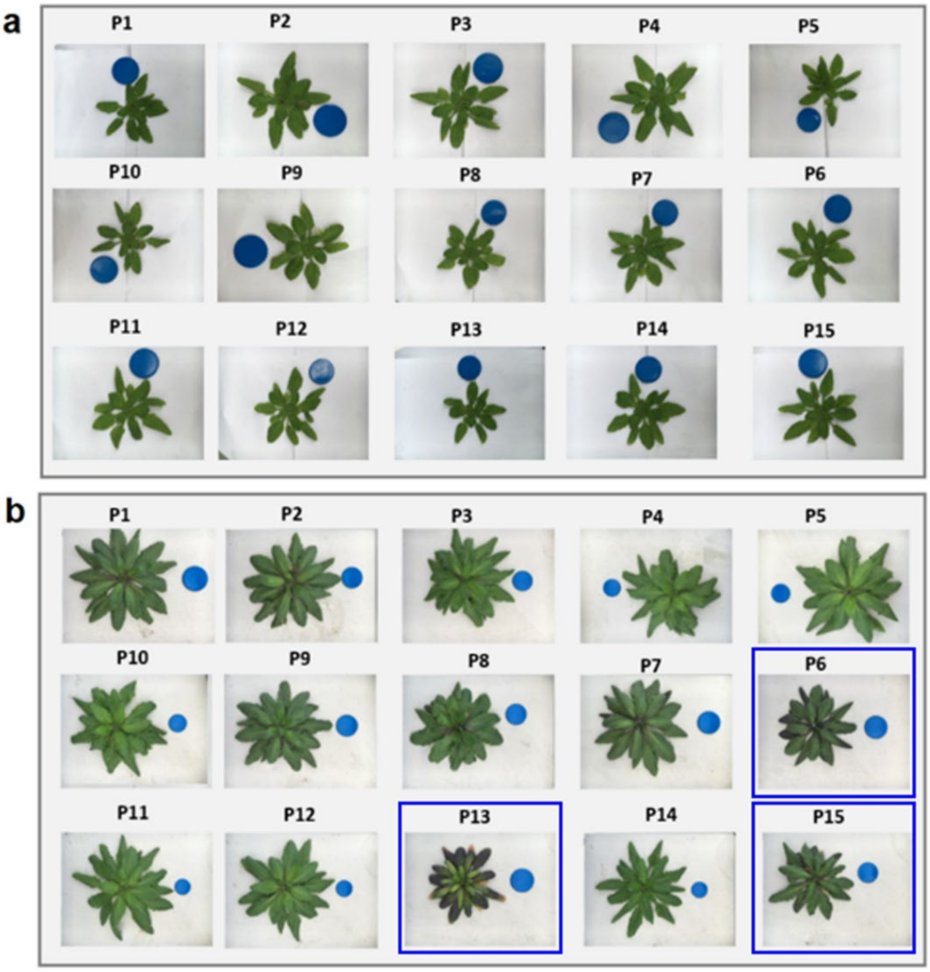

Fig. A3. 15 Arabidopsis plants before and after 7-d exposure to $^{13}CO_2$ in the labeling chamber. **a** 15 plants of similar size (PLA 14–16 cm^2) were selected and placed in the labeling chamber at the end of the light period of day 0 (the day before starting $^{13}CO_2$ treatment). **b** The same 15 plants after 7-d $^{13}CO_2$ exposure, in the early morning of day 8. The plants in blue frames had less ^{13}C incorporation in pigments. The conditions inside the labeling chamber are shown in Additional file 1; Figs. A5–A7. Blue reference chips in the images have a diameter of 2 cm. The rosettes were harvested and shock-frozen in liquid N_2 immediately after taking the images in **b**.

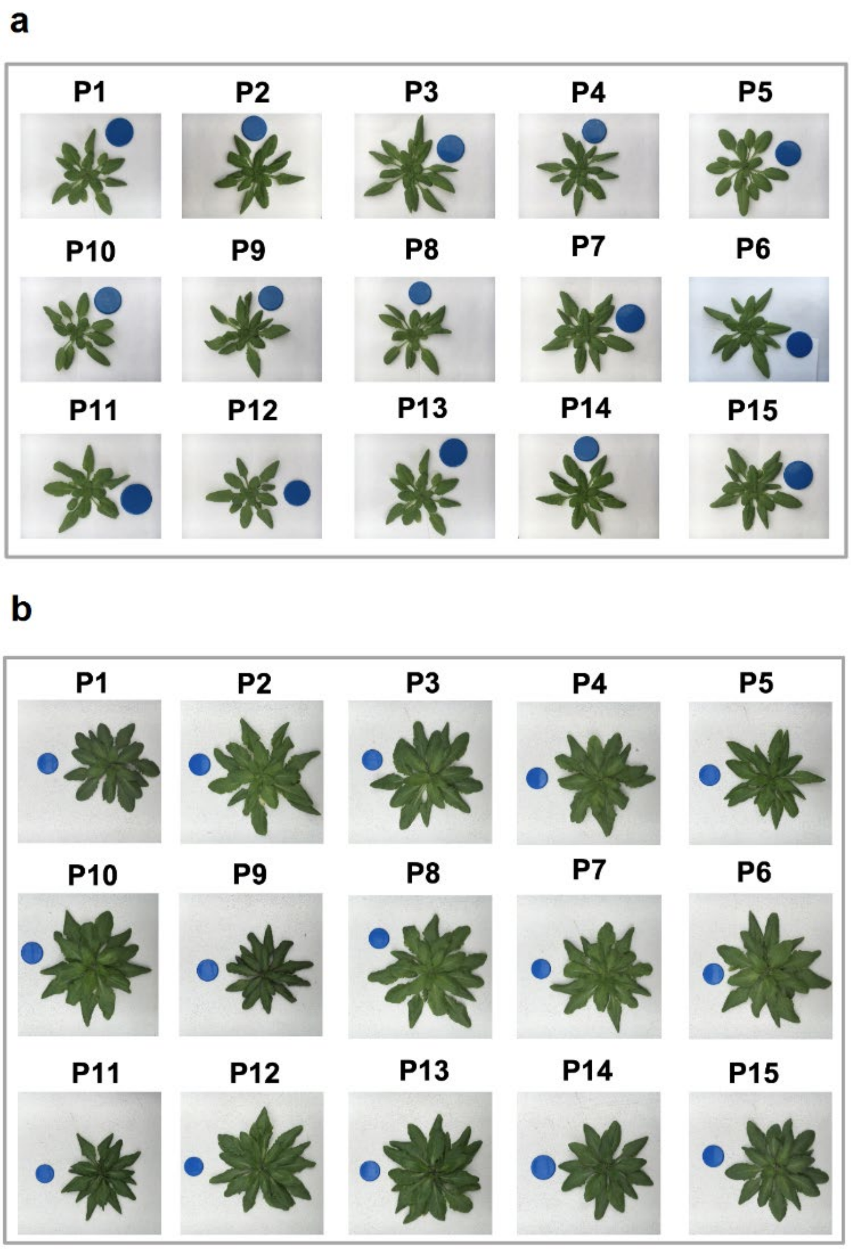

Fig. A4. 15 Arabidopsis plants before and after 7-d exposure to normal CO_2 in the labeling chamber. **a** 15 plants of similar size (PLA 14–16 cm^2) were selected and placed in the labeling chamber at the end of the light period of day 0. **b** The same 15 plants in the early morning of day 8. Blue reference chips in the images have a diameter of 2 cm.

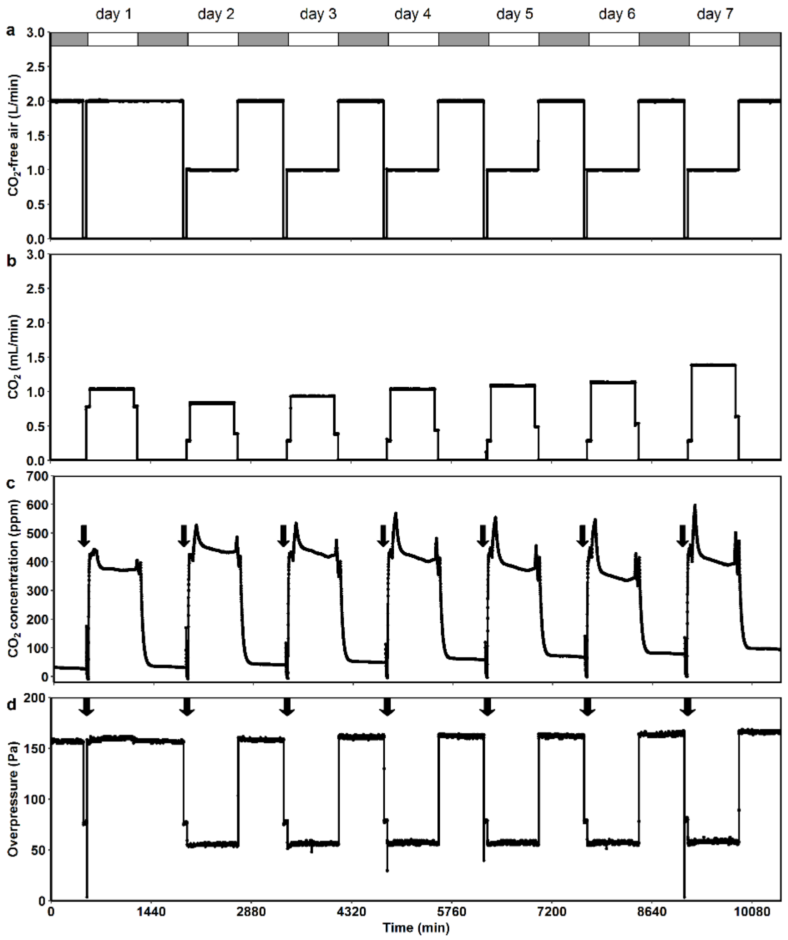

Fig. A5. Flow rates of CO_2-free air and CO_2 as well as real-time readings of the CO_2 concentration and overpressure inside the labeling chamber. **a** During the light period the flow rate of CO_2-free air (controlled by MFC3; see Fig. 1 for gas flow assembly) was set to 2 L min^{-1} on the 1st day of labeling (day 1) followed by a reduction to 1 L min^{-1} starting from day 2. The flow rate during the dark period was kept at 2 L min^{-1}. At the end of each dark period the labeling chamber was flushed with CO_2-free air (10 L min^{-1} for 45 min by MFC1) to replace the air containing CO_2 that partly came from dark respiration (indicated by arrows in **c** and **d**). This was followed by 20-min equilibration with a fresh mixture of CO_2-free air (10 L min^{-1} by MFC1) and CO_2 (5 mL min^{-1} by MFC4) before LED lamps were turned on (not shown). An outlet valve was automatically opened during flushing and equilibration. White and grey bars at the top show light and dark periods, respectively. **b** CO_2 was continuously injected into the chamber by MFC2 during the light period. Except on day 1 when a slightly higher CO_2 flow rate was applied to match the higher daytime flow rate of CO_2-free air, the initial CO_2 flow rate under morning dim light (~28 μmol photon m^{-2} s^{-1}; see Additional file 1; Fig. A7a for light intensity regime) was set to 0.3 mL min^{-1}. As with the increase in light intensity, the CO_2 flow rate was raised to 0.75 mL min^{-1} on day 2 and up to 1.5 mL min^{-1} on day 7; the flow rate had to be increased from day to day as the 15 Arabidopsis plants grew larger. When the light intensity decreased at the end of the light period, the CO_2 flow rate was also reduced to reach zero at the onset of the dark period. **c** The [CO_2] inside the labeling chamber was continuously monitored by LI-840. The daytime [CO_2] stayed at ~400 ppm except for a brief overshoot in the morning, while the nighttime [CO_2] (i.e., when the chamber was running with CO_2-free air) gradually increased from ~30 to ~100 ppm over the seven days. The daily flushing (black arrows) effectively reset [CO_2] to almost zero at the end of each dark period. **d** The labeling chamber was run under small overpressure to prevent diffusion of external ambient air into the labeling chamber and to minimize the impact of root and soil respiration on the gas inside the labeling chamber. The overpressure, which was created by the flow of CO_2-free air, was switching between ~60 Pa (when MFC3 was running at 1 L min^{-1}) and 160–170 Pa (when it was running at 2 L min^{-1}). The overpressure was ~80 Pa during the flushing and equilibration (black arrows).

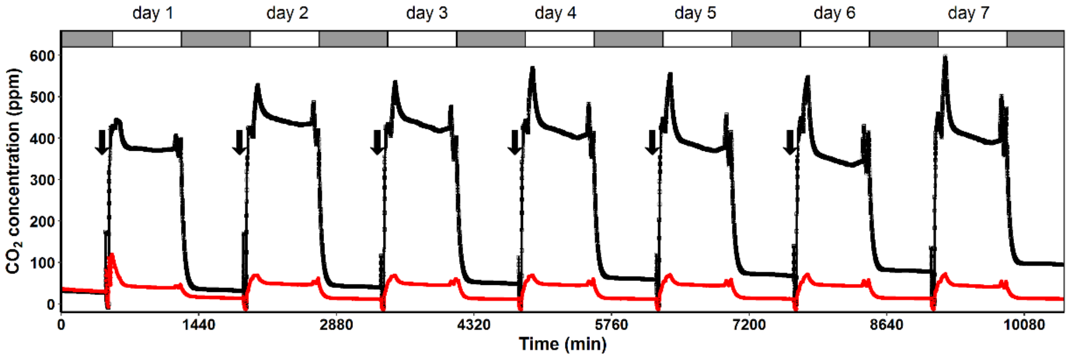

Fig. A6. CO_2 concentration measured by LI-840. The labeling chamber was running with normal CO_2 (black line) or $^{13}CO_2$ (red line). Note that LI-840 has a very low sensitivity to $^{13}CO_2$ and thus shows very low values in $^{13}CO_2$. Nevertheless, the flow rate program (see Additional file 1; Fig. A5a, b) produced highly reproducible light/dark patterns of [CO_2] in both CO_2 and $^{13}CO_2$ conditions. Black arrows show flushing and equilibration events at the end of each dark period. White and grey bars at the top are for light and dark periods, respectively.

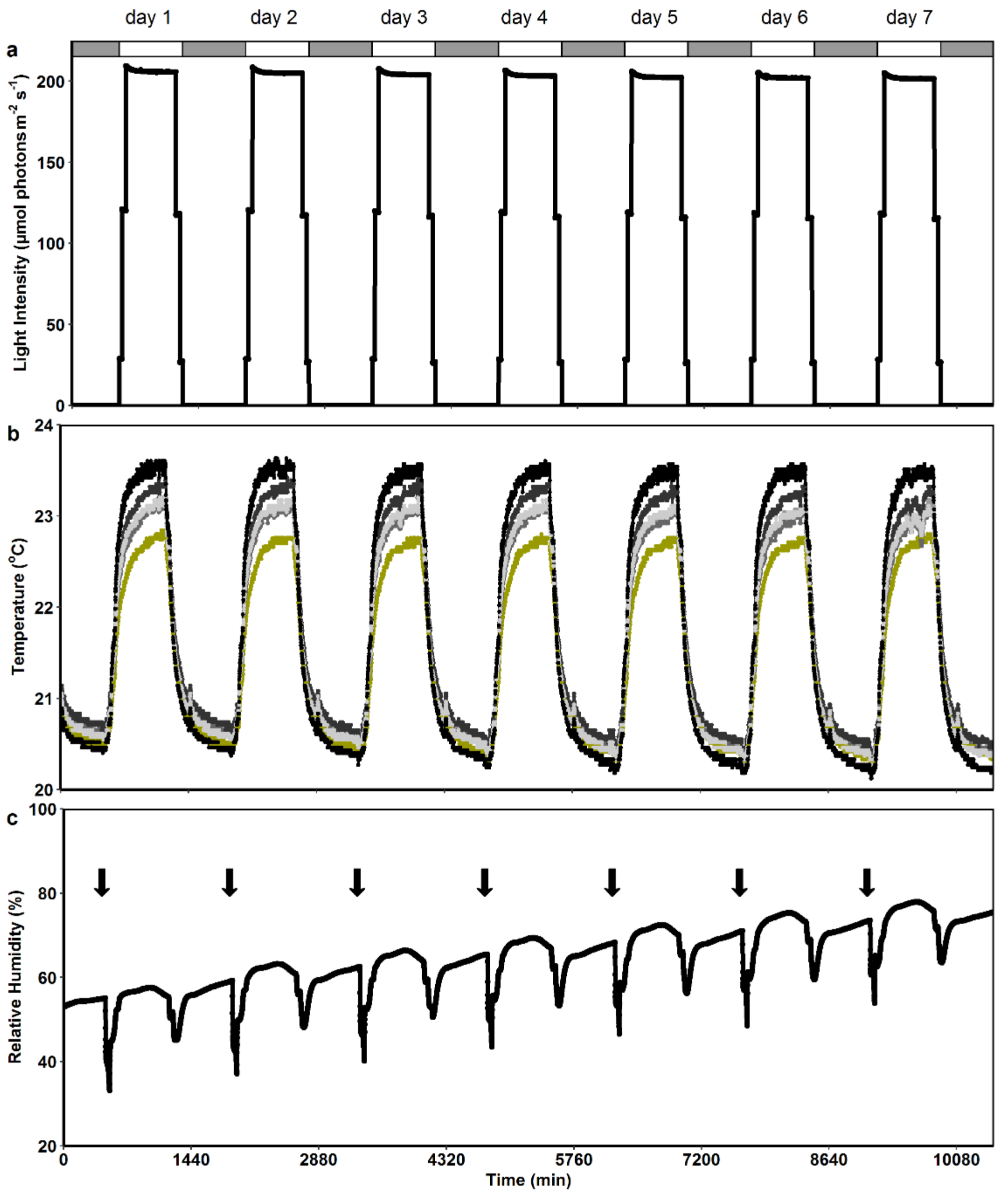

Fig. A7. Conditions inside the $^{13}CO_2$ labeling chamber. **a** The light intensity was measured at the center of the closed labeling chamber (see Fig. 1 for sensor positions). The intensity of the LED lamps was increased or decreased stepwise at the beginning or at the end of the light period, respectively. The white and grey bars at the top show 12 h/12 h light/dark regime. **b** The temperature was measured by five temperature sensors as well as a temperature probe of the humidity sensor mounted at different positions of the labeling chamber. While the nighttime temperature was around 20.5°C in all positions, the daytime values under the LED lamps varied between 22.5 and 23.5°C. Note that the temperature sensors (except the temperature probe of the humidity sensor; green line) were directly exposed to the light, which may explain their higher readings. **c** The relative air humidity in the closed labeling chamber gradually increased from ~55% to ~75% over seven day/night cycles as the plants grew larger and transpired more. The black arrows show flushing and equilibration events.

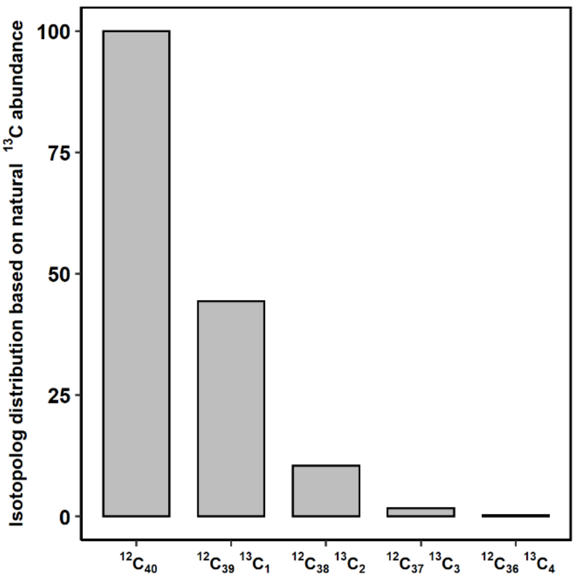

Fig. A8. Simulation of carotenoid isotopolog distribution based on the natural ^{13}C abundance.

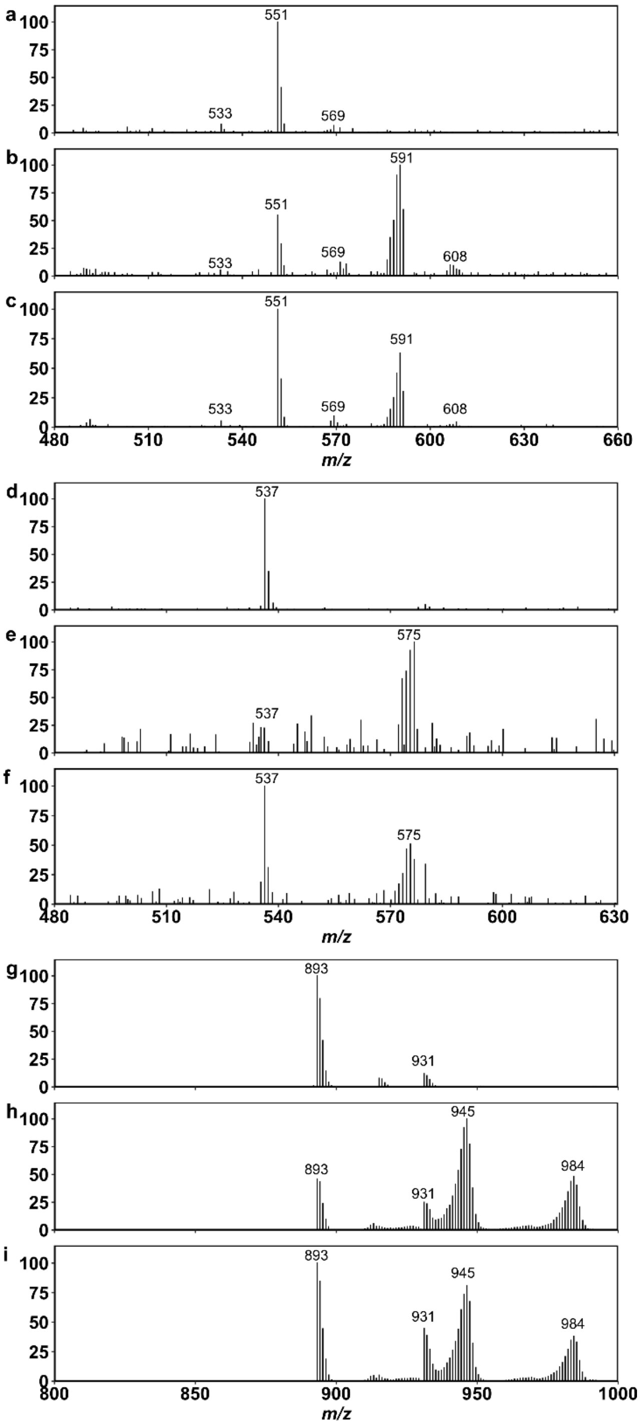

Fig. A9. Mass spectra of Lut (**a**–**c**), all-*trans*-β-Car (**d**–**f**) and Chl *a* (**g**–**i**) from the spike tests of TQ-MS. **a**, **d** and **g** Non-labeled Lut, β-Car and Chl *a* standard diluted in acetone. **b**, **e** and **h** Lut, β-Car and Chl *a* of a ^{13}C-labeled Arabidopsis leaf pigment extract. **c**, **f** and **i** Non-labeled Lut, β-Car and Chl *a* standard (shown in **a**, **d** and **g**) added to the ^{13}C-labeled Arabidopsis leaf pigment extract (shown in **b**, **e** and **h**). The pigment recovery is summarized in Additional file 2; Table A1.

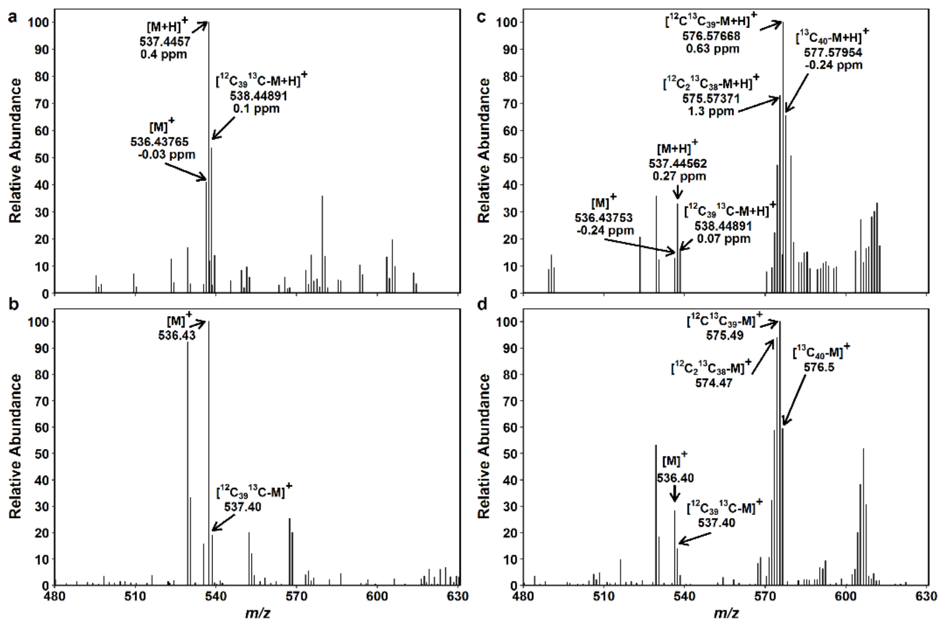

Fig. A10. Mass spectra of 9-*cis*-β-Car extracted from non-labeled and ^{13}C-labeled Arabidopsis plants. FTICR-MS showing $[M]^+$ and $[M+H]^+$ ions of a non-labeled (**a**) and a ^{13}C-labeled (**c**) sample. Deviations from the expected mass (Δ) are given in parts per million (ppm). TQ-MS showing predominantly $[M]^+$ ion in the same non-labeled (**b**) and ^{13}C-labeled (**d**) samples as in **a** and **c**. Overlapping mass peaks of $[M]^+$ and $[M+H]^+$ ions are regarded as $[M+H]^+$ or $[M]^+$ in the analysis of FTICR-MS and TQ-MS, respectively. Peak assignment is according to Additional file 2; Tables A3–A6.

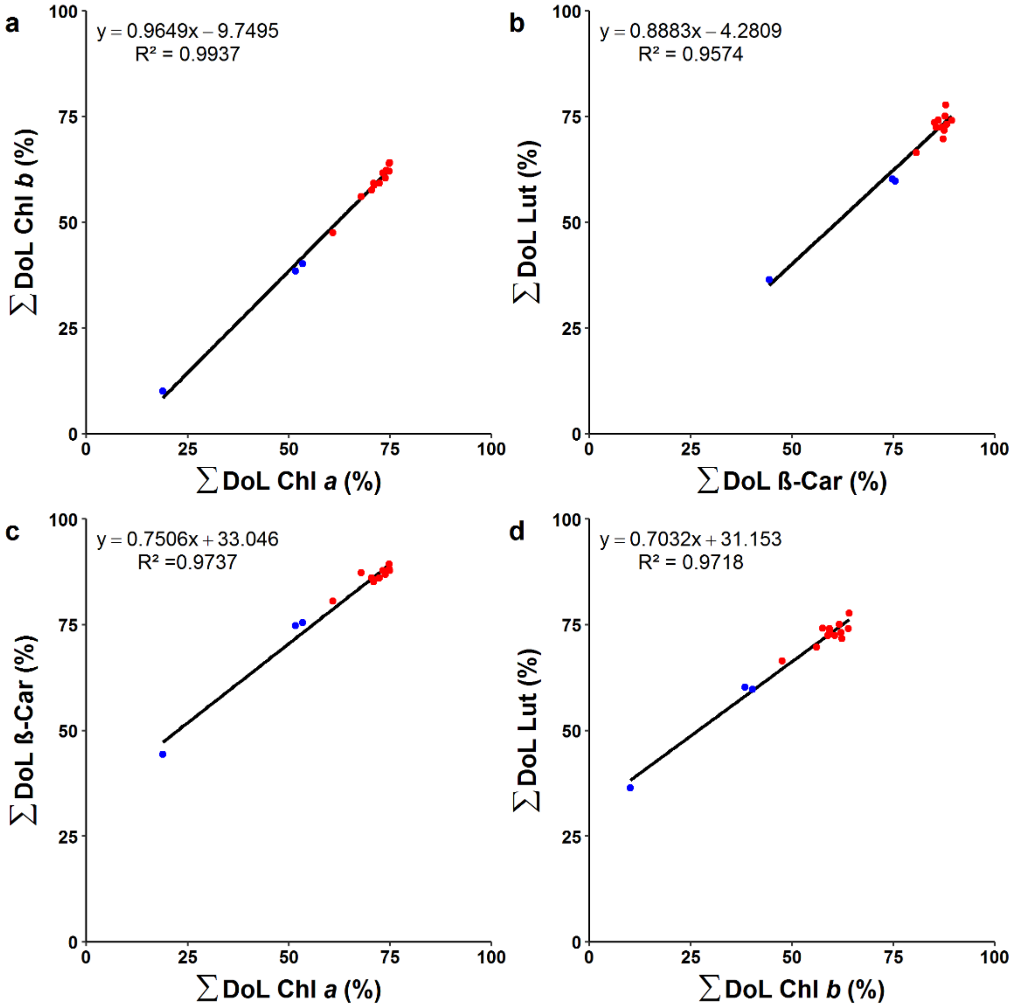

Fig. A11. Correlation between the degree of ^{13}C labeling (ΣDoL) of different pigments extracted from Arabidopsis leaves after 7-d $^{13}CO_2$ labeling. **a** Chl *a* and Chl *b*. **b** All-*trans*-β-Car and Lut. **c** Chl *a* and all-*trans*-β-Car. **d** Chl *b* and Lut. Red and blue symbols represent plants that had higher (n=12) or lower (n=3) ^{13}C incorporation in pigments, respectively.
