# Supplementary material for: Combination of long-term 13CO2 labeling and isotopolog profiling allows turnover analysis of photosynthetic pigments in Arabidopsis leaves
Source: Plant Methods. 2022 Oct 1;18:114. doi: 10.1186/s13007-022-00946-3 (PMC9526918; doi:10.1186/s13007-022-00946-3)
Supplement: Supplementary file 2 — Additional file 2: Table S1. Pigment recovery of all-trans-β-Car, Lut and Chl a. Table S2. Reproducibility of ΣDoL and NLP calculated from TQ-MS data. Table S3. Peak assignment and calculation of BPIi, BPIi(norm) and DoLi for individual isotopologs of all-trans-β-Car from a non-labeled Arabidopsis plant obtained by FTICR-MS. Table S4. Peak assignment and calculation of BPIi, BPIi(norm) and DoLi for individual isotopologs of all-trans-β-Car from a non-labeled Arabidopsis plant obtained by TQ-MS. Table S5. Peak assignment and calculation of BPIi, BPIi(norm) and DoLi for individual isotopologs of all-trans-β-Car from a 13C-labeled Arabidopsis plant obtained by FTICR-MS. Table S6. Peak assignment and calculation of BPIi, BPIi(norm) and DoLi for individual isotopologs of all-trans-β-Car from a 13C-labeled Arabidopsis plant obtained by TQ-MS. Table S7. Peak assignment and calculation of BPIi, BPIi(norm) and DoLi for individual isotopologs of Lut from a non-labeled Arabidopsis plant obtained by FTICR-MS. Table S8. Peak assignment and calculation of BPIi, BPIi(norm) and DoLi for individual isotopologs of Lut from a non-labeled Arabidopsis plant obtained by TQ-MS. Table S9. Peak assignment and calculation of BPIi, BPIi(norm) and DoLi for individual isotopologs of Lut from a 13C-labeled Arabidopsis plant obtained by FTICR-MS. Table S10. Peak assignment and calculation of BPIi, BPIi(norm) and DoLi for individual isotopologs of Lut from a 13C-labeled Arabidopsis plant obtained by TQ-MS. Table S11. Peak assignment and calculation of BPIi, BPIi(norm) and DoLi for individual isotopologs of Chl a from a non-labeled Arabidopsis plant obtained by FTICR-MS. Table S12. Peak assignment and calculation of BPIi, BPIi(norm) and DoLi for individual isotopologs of Chl a from a non-labeled Arabidopsis plant obtained by TQ-MS. Table S13. Peak assignment and calculation of BPIi, BPIi(norm) and DoLi for individual isotopologs of Chl a from a 13C-labeled Arabidopsis plant obtained by FTICR-MS. Ta [file 13007_2022_946_MOESM2_ESM.pdf]

Additional file 2

Table A1. Pigment recovery of all-*trans*-β-Car, Lut and Chl *a* was checked by adding non-labeled pigment standards of known concentrations to a ^{13}C-labeled Arabidopsis leaf sample during pigment extraction. Changes in non-labeled population (NLP) with and without spike were analyzed to calculate the recovery. For B and C, pigment contents were calculated from the concentrations per injection (in ng; determined by LC) and the size of NLP (in %; determined by TQ-MS). For mass spectra of the spike tests, see Additional file 1; Fig. A9.

(ng)	A: non-labeled pigment standard	B: NLP measured in ^{13}C-labeled leaf pigment sample	C: NLP measured in ^{13}C-labeled leaf pigment sample (B) spiked with non-labeled standard (A)	D: C – B	Recovery: D / A
β-Car	18.1	2.36	19.76	17.39	0.961
Lut	16.9	6.97	23.07	16.10	0.953
Chl *a*	67.9	29.25	93.77	64.52	0.950

Table A2. Reproducibility of ΣDoL and NLP calculated from TQ-MS data. The ΣDoL and NLP values are means ±SD of three (all-*trans*-β-Car, Lut and Chl *a*) or four (Chl *b*) repeated injections of a ^{13}C-labeled Arabidopsis leaf pigment sample.

(%)	β-Car	Lut	Chl *a*	Chl *b*
ΣDoL	87.36 ±0.76	74.50 ±1.90	73.70 ±0.92	63.00 ±1.08
NLP	9.25 ±0.78	21.81 ±2.05	15.07 ±0.66	28.08 ±1.35

Table A3. Peak assignment and calculation of base peak intensity (BPI_i), normalized BPI_i ($BPI_{i(norm)}$) and degree of ^{13}C labeling (DoL_i) for individual isotopologs of all-*trans*-β-Car from a non-labeled Arabidopsis plant obtained by FTICR-MS. $[M+H]^+$ is the predominant quasi-molecular ion of β-Car in our FTICR-MS data. The mass peaks of $[M]^+$ and $[M+H]^+$ ions are overlapping at *m*/*z* 537–539 ($^{\#}$). Taking into account the contributions of $[M]^+$ and $[M+H]^+$ in this *m*/*z* region based on the theoretical isotopolog distribution of carotenoid (see Additional file 1; Fig. A8) results in an increase in ΣDoL by 0.2 points (from 1.15 to 1.35). The mass spectrum of this sample is shown in Fig. 5a. *i* gives the number of ^{13}C atom in the molecule.

m/z	**Intensity**	**Δ** (ppm)	**Empirical formula**	**Quasi-molecular ion**	**BPI*i***	**BPI*i* (norm)** (%)	***i***	**DoL*i*** (%)
536.43765	62654.8	0.00	$^{12}C_{40}H_{56}$	$[M]^+$	29.34	14.64	0	0.00
537.44556$^{\#}$	213524.9	0.16	$^{12}C_{40}H_{57}$	$[M+H]^+$	100.00	49.89	0	0.00
538.44887$^{\#}$	112848.8	0.07	$^{12}C_{39}{}^{13}CH_{57}$		52.85	26.37	1	0.66
539.45223$^{\#}$	33080.8	0.08	$^{12}C_{38}{}^{13}C_2H_{57}$		15.49	7.73	2	0.39
540.45548	5868.2	-0.12	$^{12}C_{37}{}^{13}C_3H_{57}$		2.75	1.37	3	0.10
				Total	200.43	100.00	**ΣDoL**	**1.15**

$^{\#}$ Overlapping mass peaks. Empirical formulae and **Δ** are for the predominant $[M+H]^+$ ion.

Table A4. Peak assignment and calculation of BPI_i, $BPI_{i(norm)}$ and DoL_i for individual isotopologs of all-*trans*-β-Car from a non-labeled Arabidopsis plant obtained by TQ-MS. $[M]^+$ is the predominant molecular ion of β-Car in our TQ-MS data. The overlapping mass peaks of $[M]^+$ and $[M+H]^+$ ions, which are not separated at *m*/*z* 537–539 ($^{\#}$), are regarded as $[M]^+$. The mass spectrum of this sample is shown in Fig. 5b. *i* gives the number of ^{13}C atom in the molecule.

m/z	**Intensity**	**Quasi-molecular ion**	**BPI*i***	**BPI*i* (norm)** (%)	***i***	**DoL*i*** (%)
536.44	2.62E+07	$[M]^+$	100.00	53.29	0	0.00
537.4$^{\#}$	1.87E+07		71.60	38.15	1	0.95
538.55$^{\#}$	3.91E+06		14.93	7.95	2	0.40
539.54$^{\#}$	2.95E+05		1.13	0.60	3	0.04
		Total	186.66	100.00	**ΣDoL**	**1.40**

$^{\#}$ Overlapping mass peaks.

Table A5. Peak assignment and calculation of BPI_i, $BPI_{i(norm)}$ and DoL_i for individual isotopologs of all-*trans*-β-Car from a ^{13}C-labeled Arabidopsis plant obtained by FTICR-MS. The contribution of $[M]^+$ ion in the overlapping *m*/*z* region is very minor in ^{13}C-labeled samples so that calculation by taking into account the contributions of $[M]^+$ and $[M+H]^+$ at *m*/*z* 537–538 ($^{\#}$) results in a marginal increase in ΣDoL by 0.09 points (from 84.16 to 84.25). FTICR-MS can separate the mass peaks of ^{13}C-labeled $[M]^+$ and $[M+H]^+$ at *m*/*z* 570–575 (*). White and shaded cells denote non-labeled (i.e., not labeled beyond the natural abundance of ^{13}C) and ^{13}C-labeled isotopologs of all-*trans*-β-Car, respectively. The mass spectrum of this sample is shown in Fig. 5c. *i* gives the number of ^{13}C atom in the molecule.

m/z	**Intensity**	**Δ** (ppm)	**Empirical formula**	**Quasi-molecular ion**	**BPI*i***	**BPI*i*(norm)** (%)	***i***	**DoL*i*** (%)
536.43787	21225.0	0.40	$^{12}C_{40}H_{56}$	$[M]^+$	7.33	1.82	0	0.00
537.44587$^{\#}$	74723.0	0.73	$^{12}C_{40}H_{57}$	$[M+H]^+$	25.81	6.40	0	0.00
538.44919$^{\#}$	37552.3	0.67	$^{12}C_{39}{}^{13}CH_{57}$		12.97	3.21	1	0.08
539.45245	8499.9	0.48	$^{12}C_{38}{}^{13}C_2H_{57}$		2.94	0.73	2	0.04
570.55013*	1214.0	-2.77	$^{12}C_6{}^{13}C_{34}H_{56}$	$[M]^+$	0.42	0.10	34	0.09
571.5538*	5250.3	-2.22	$^{12}C_5{}^{13}C_{35}H_{56}$		1.81	0.45	35	0.39
572.55693*	7354.2	-2.61	$^{12}C_4{}^{13}C_{36}H_{56}$		2.54	0.63	36	0.57
574.56309*	33013.8	-3.56	$^{12}C_2{}^{13}C_{38}H_{56}$		11.40	2.83	38	2.68
575.56661*	41046.0	-3.27	$^{12}C\ {}^{13}C_{39}H_{56}$		14.17	3.51	39	3.42
569.55406	3797.3	2.16	$^{12}C_8{}^{13}C_{32}H_{57}$	$[M+H]^+$	1.31	0.32	32	0.26
570.55780*	4145.9	2.83	$^{12}C_7{}^{13}C_{33}H_{57}$		1.43	0.35	33	0.29
571.56140*	8791.5	3.25	$^{12}C_6{}^{13}C_{34}H_{57}$		3.04	0.75	34	0.64
572.56424*	26620.1	2.35	$^{12}C_5{}^{13}C_{35}H_{57}$		9.19	2.28	35	1.99
573.5673*	64647.0	1.82	$^{12}C_4{}^{13}C_{36}H_{57}$		22.33	5.53	36	4.98
574.57075*	124218.2	1.99	$^{12}C_3{}^{13}C_{37}H_{57}$		42.90	10.63	37	9.83
575.57392*	220249.5	1.67	$^{12}C_2{}^{13}C_{38}H_{57}$		76.06	18.85	38	17.91
576.57694$^{\#}$	289567.2	1.08	$^{12}C^{13}C_{39}H_{57}$		100.00	24.78	39	24.16
577.57986	196470.0	0.33	$^{13}C_{40}H_{57}$		67.85	16.82	40	16.82
				Total	403.5	100.00	**ΣDoL**	**84.16**

$^{\#}$ Overlapping mass peaks. Empirical formulae and **Δ** are for the predominant $[M+H]^+$ ion.

* Overlapping mass peaks in TQ-MS that are separated by FTICR-MS.

Table A6. Peak assignment and calculation of BPI_i, $BPI_{i(norm)}$ and DoL_i for individual isotopologs of all-*trans*-β-Car from a ^{13}C-labeled Arabidopsis plant obtained by TQ-MS. The calculation does not take into account $[M+H]^+$ ion in the overlapping *m*/*z* regions ($^{\#}$) of both non-labeled and ^{13}C-labeled populations. Thus, ΣDoL increases by 1.89 points by TQ-MS compared to FTICR-MS that can separate the mass peaks of ^{13}C-labeled $[M]^+$ and $[M+H]^+$ at *m*/*z* 570–576 (cf. Additional file 2; Table A5). White and shaded cells denote non-labeled and ^{13}C-labeled isotopologs of all-*trans*-β-Car, respectively. Note that the overlaps of mass peaks do not affect the relative abundance of non-labeled and ^{13}C-labeled populations since they occur only within each population. The mass spectrum of this sample is shown in Fig. 5d. *i* gives the number of ^{13}C atom in the molecule.

m/z	**Intensity**	**Quasi-molecular ion**	**BPI*i***	**BPI*i* (norm)** (%)	***i***	**DoL*i*** (%)
536.41	5.05E+06	$[M]^+$	25.55	6.91	0	0.00
537.4$^{\#}$	2.25E+06		11.40	3.08	1	0.08
538.52	2.18E+05		1.10	0.30	2	0.01
571.43$^{\#}$	1.19E+06	$[M]^+$	5.99	1.62	35	1.42
572.44$^{\#}$	5.08E+06		25.68	6.95	36	6.25
573.48$^{\#}$	9.90E+06		50.09	13.56	37	12.54
574.5$^{\#}$	1.69E+07		85.33	23.09	38	21.94
575.5$^{\#}$	1.98E+07		100.00	27.06	39	26.38
576.51$^{\#}$	1.27E+07		64.39	17.42	40	17.42
		Total	369.53	100.00	**ΣDoL**	**86.05**

$^{\#}$ Overlapping mass peaks.

Table A7. Peak assignment and calculation of BPI_i, $BPI_{i(norm)}$ and DoL_i for individual isotopologs of Lut from a non-labeled Arabidopsis plant obtained by FTICR-MS. $[M+H–H_2O]^+$ is the predominant quasi-molecular ion of Lut in the FTICR-MS data. The mass spectrum of this sample is shown in Fig. 6a. *i* gives the number of ^{13}C atom in the molecule.

m/z	**Intensity**	**Δ** (ppm)	**Empirical formula**	**Quasi-molecular ion**	**BPI**	$BPI_{i(norm)}$ (%)	***i***	DoL_i (%)
533.41412	31429.3	-0.10	$^{12}C_{40}H_{53}$	$[M+H–2H_2O]^+$	6.22	2.90	0	0.00
534.4176	17021.6	0.12	$^{12}C_{39}{}^{13}CH_{53}$		3.37	1.57	1	0.04
535.42042	2840.4	-0.87	$^{12}C_{38}{}^{13}C_2H_{53}$		0.56	0.26	2	0.01
551.42474	505217.5	0.00	$^{12}C_{40}H_{55}O$	$[M+H–H_2O]^+$	100.00	46.68	0	0.00
552.42814	271674.0	0.08	$^{12}C_{39}{}^{13}CH_{55}O$		53.77	25.10	1	0.63
553.43146	74532.6	0.01	$^{12}C_{38}{}^{13}C_2H_{55}O$		14.75	6.89	2	0.34
554.43493	15213.1	0.22	$^{12}C_{37}{}^{13}C_3H_{55}O$		3.01	1.41	3	0.11
568.42756	61215.8	0.13	$^{12}C_{40}H_{56}O_2$	$[M]^+$	12.12	5.66	0	0.00
569.42893*	22825.7	-3.35	$^{12}C_{39}{}^{13}CH_{56}O_2$		4.52	2.11	1	0.05
570.43199*	7436.1	-3.85	$^{12}C_{38}{}^{13}C_2H_{56}O_2$		1.47	0.69	2	0.03
569.43629*	40965.7	1.73	$^{12}C_{40}H_{57}O_2$	$[M+H]^+$	8.11	3.79	0	0.00
570.43928*	24101.5	1.08	$^{12}C_{39}{}^{13}CH_{57}O_2$		4.77	2.23	1	0.06
571.44263	5976.4	1.08	$^{12}C_{38}{}^{13}C_2H_{57}O_2$		1.18	0.55	2	0.03
572.44484	1854.0	-0.94	$^{12}C_{37}{}^{13}C_3H_{57}O_2$		0.37	0.17	3	0.01
				Total	214.22	100.00	**ΣDoL**	**1.31**

* Overlapping mass peaks in TQ-MS that are separated by FTICR-MS.

Table A8. Peak assignment and calculation of BPI_i, $BPI_{i(norm)}$ and DoL_i for individual isotopologs of Lut from a non-labeled Arabidopsis plant obtained by TQ-MS. $[M+H–H_2O]^+$ is the predominant quasi-molecular ion of Lut also in TQ-MS data. The overlapping mass peaks of $[M]^+$ and $[M+H]^+$, which are not separated at *m/z* 569–570 (#), are considered $[M+H]^+$. Still, the difference in ΣDoL between FTICR-MS and TQ-MS is no more than 0.28 points (cf. Additional file 2; Table A7). The mass spectrum of this sample is shown in Fig. 6b. *i* gives the number of ^{13}C atom in the molecule.

m/z	**Intensity**	**Quasi-molecular ion**	BPI_i	$BPI_{i(norm)}$ (%)	***i***	DoL_i (%)
533.36	1.06E+06	$[M+H–2H_2O]^+$	5.53	2.71	0	0.00
534.45	7.98E+05		4.16	2.04	1	0.05
551.42	1.92E+07	$[M+H–H_2O]^+$	100.00	49.07	0	0.00
552.4	1.08E+07		56.33	27.64	1	0.69
553.52	1.35E+06		7.02	3.45	2	0.17
554.64	3.46E+05		1.80	0.88	3	0.07
568.36	1.92E+06	$[M]^+$	10.02	4.91	0	0.00
569.37#	2.83E+06	$[M+H]^+$	14.73	7.23	0	0.00
570.4#	8.06E+05		4.20	2.06	1	0.05
		Total	203.64	100.00	**ΣDoL**	**1.03**

Overlapping mass peaks.

Table A9. Peak assignment and calculation of BPI_i, $BPI_{i(\mathrm{norm})}$ and DoL_i for individual isotopologs of Lut from a ^{13}C-labeled Arabidopsis plant obtained by FTICR-MS. FTICR-MS can separate small peaks of ^{13}C-labeled $[M+H–2H_2O]^+$ and non-labelled $[M]^+$ and $[M+H]^+$ appearing at *m*/*z* 569–571 as well as ^{13}C-labeled $[M]^+$ and $[M+H]^+$ at *m*/*z* 607–608. White and shaded cells denote non-labeled and ^{13}C-labeled isotopologs of Lut, respectively. The mass spectrum of this sample is shown in Fig. 6c. *i* gives the number of ^{13}C atom in the molecule.

m/z	**Intensity**	**Δ** (ppm)	**Empirical formula**	**Quasi-molecular ion**	**BPI**	**$BPI_{i(norm)}$** (%)	***i***	**DoL_i** (%)
533.41438	46515.9	0.38	$^{12}C_{40}H_{53}$	$[M+H–2H_2O]^+$	3.68	0.68	0	0.00
534.41791	14708.1	0.70	$^{12}C_{39}{}^{13}CH_{53}$		1.17	0.21	1	0.01
551.42498	787922.4	0.43	$^{12}C_{40}H_{55}O$	$[M+H–H_2O]^+$	62.42	11.44	0	0.00
552.42832	342399.1	0.40	$^{12}C_{39}{}^{13}CH_{55}O$		27.12	4.97	1	0.12
553.43169	74819.1	0.42	$^{12}C_{38}{}^{13}C_2H_{55}O$		5.93	1.09	2	0.05
554.43479	19652.7	-0.03	$^{12}C_{37}{}^{13}C_3H_{55}O$		1.56	0.29	3	0.02
568.42801	70533.8	0.94	$^{12}C_{40}H_{56}O_2$	$[M]^+$	5.59	1.02	0	0.00
569.42875*	12686.4	-3.66	$^{12}C_{39}{}^{13}CH_{56}O_2$		1.00	0.18	1	0.00
569.43651*	43644.5	2.12	$^{12}C_{40}H_{57}O_2$	$[M+H]^+$	3.46	0.63	0	0.00
570.4392*	27536.8	0.95	$^{12}C_{39}{}^{13}CH_{57}O_2$		2.18	0.40	1	0.01
571.44235*	7388.3	0.58	$^{12}C_{38}{}^{13}C_2H_{57}O_2$		0.59	0.11	2	0.01
567.52853	5600.6	0.50	$^{12}C_6{}^{13}C_{34}H_{53}$	$[M+H–2H_2O]^+$	0.08	0.08	34	0.07
568.53228	8412.7	1.21	$^{12}C_5{}^{13}C_{35}H_{53}$		0.12	0.12	35	0.11
569.53497*	15126.5	0.03	$^{12}C_4{}^{13}C_{36}H_{53}$		1.20	0.22	36	0.20
570.53853*	25304.5	0.39	$^{12}C_3{}^{13}C_{37}H_{53}$		2.00	0.37	37	0.34
571.54201*	44291.7	0.60	$^{12}C_2{}^{13}C_{38}H_{53}$		3.51	0.64	38	0.61
572.54507	51306.3	0.10	$^{12}C{}^{13}C_{39}H_{53}$		4.06	0.75	39	0.73
573.54862	26589.2	0.43	$^{13}C_{40}H_{53}$		2.11	0.39	40	0.39
578.5154	7447.5	0.13	$^{12}C_{13}{}^{13}C_{27}H_{55}O$	$[M+H–H_2O]^+$	0.59	0.11	27	0.07
579.51791	7445.8	-1.32	$^{12}C_{12}{}^{13}C_{28}H_{55}O$		0.59	0.11	28	0.08
580.52211	15008.8	0.14	$^{12}C_{11}{}^{13}C_{29}H_{55}O$		1.19	0.22	29	0.16
581.52572	17330.1	0.56	$^{12}C_{10}{}^{13}C_{30}H_{55}O$		1.37	0.25	30	0.19
582.5288	23840.1	0.09	$^{12}C_9{}^{13}C_{31}H_{55}O$		1.89	0.35	31	0.27
583.53218	41403.4	0.14	$^{12}C_8{}^{13}C_{32}H_{55}O$		3.28	0.60	32	0.48
584.5357	56495.1	0.42	$^{12}C_7{}^{13}C_{33}H_{55}O$		4.48	0.82	33	0.68
585.53907	102032.3	0.44	$^{12}C_6{}^{13}C_{34}H_{55}O$		8.08	1.48	34	1.26
586.54242	200844.8	0.44	$^{12}C_5{}^{13}C_{35}H_{55}O$		15.91	2.92	35	2.55
587.54576	384374.8	0.42	$^{12}C_4{}^{13}C_{36}H_{55}O$		30.45	5.58	36	5.02
588.54909	707973.1	0.38	$^{12}C_3{}^{13}C_{37}H_{55}O$		56.08	10.28	37	9.51
589.55241	1107607.0	0.32	$^{12}C_2{}^{13}C_{38}H_{55}O$		87.74	16.09	38	15.28
590.55574	1262336.0	0.27	$^{12}C{}^{13}C_{39}H_{55}O$		100.00	18.33	39	17.87
591.55917	715246.8	0.41	$^{13}C_{40}H_{55}O$		56.66	10.39	40	10.39
599.53163	4504.1	0.25	$^{12}C_9{}^{13}C_{31}H_{56}O_2$	$[M]^+$	0.36	0.07	31	0.05
602.54187	8899.8	0.54	$^{12}C_6{}^{13}C_{34}H_{56}O_2$		0.71	0.13	34	0.11
603.54471	12384.4	-0.32	$^{12}C_5{}^{13}C_{35}H_{56}O_2$		0.98	0.18	35	0.16
604.5482	34569.2	-0.09	$^{12}C_4{}^{13}C_{36}H_{56}O_2$		2.74	0.50	36	0.45
605.55141	38808.2	-0.34	$^{12}C_3{}^{13}C_{37}H_{56}O_2$		3.07	0.56	37	0.52
606.55443	80000.4	-0.88	$^{12}C_2{}^{13}C_{38}H_{56}O_2$		6.34	1.16	38	1.10
607.55685*	88651.6	-2.42	$^{12}C{}^{13}C_{39}H_{56}O_2$		7.02	1.29	39	1.26
608.55888*	45066.4	-4.58	$^{13}C_{40}H_{56}O_2$		3.57	0.65	40	0.65
607.56532*	90565.0	4.16	$^{12}C_2{}^{13}C_{38}H_{57}O_2$	$[M+H]^+$	7.17	1.32	38	1.25
608.56778*	111631.7	2.68	$^{12}C{}^{13}C_{39}H_{57}O_2$		8.84	1.62	39	1.58
609.56987	96633.5	0.60	$^{13}C_{40}H_{57}O_2$		7.66	1.40	40	1.40
				Total	544.35	100.00	**ΣDoL**	**75.01**

* Overlapping mass peaks in TQ-MS that are separated by FTICR-MS.

Table A10. Peak assignment and calculation of BPI_i, $BPI_{i(norm)}$ and DoL_i for individual isotopologs of Lut from a ^{13}C-labeled Arabidopsis plant obtained by TQ-MS. The overlapping mass peaks of non-labeled $[M]^+$ and $[M+H]^+$ at *m/z* 569–570 (#) and ^{13}C-labeled $[M]^+$ and $[M+H]^+$ at *m/z* 607–608 (#) are regarded as $[M+H]^+$. White and shaded cells denote non-labeled and ^{13}C-labeled isotopologs of Lut, respectively. The mass spectrum of this sample is shown in Fig. 6d. *i* gives the number of ^{13}C atom in the molecule.

m/z	**Intensity**	**Quasi-molecular ion**	**BPI**	**BPI*i* (norm) (%)**	***i***	**DoL*i* (%)**
533.36	4.42E+05	$[M+H–2H_2O]^+$	4.99	0.85	0	0.00
534.36	4.19E+05		4.74	0.81	1	0.02
551.39	5.54E+06	$[M+H–H_2O]^+$	62.64	10.66	0	0.00
552.39	2.85E+06		32.19	5.48	1	0.14
553.43	3.07E+05		3.47	0.59	2	0.03
568.31	5.11E+05	$[M]^+$	5.78	0.98	0	0.00
569.33#	1.01E+06	$[M+H]^+$	11.47	1.95	0	0.00
570.4#	2.91E+05		3.29	0.56	1	0.01
571.47	2.84E+04		0.32	0.05	2	0.00
572.38	3.27E+05	$[M+H–2H_2O]^+$	3.70	0.63	39	0.61
573.33	3.10E+05		3.51	0.60	40	0.60
578.4	2.74E+04	$[M+H–H_2O]^+$	0.31	0.05	27	0.04
579.25	2.63E+04		0.30	0.05	28	0.04
580.37	2.17E+04		0.25	0.04	29	0.03
581.39	1.31E+05		1.48	0.25	30	0.19
583.33	3.09E+05		3.49	0.59	32	0.48
584.41	3.55E+05		4.01	0.68	33	0.56
585.38	7.78E+05		8.80	1.50	34	1.27
586.43	1.46E+06		16.48	2.80	35	2.45
587.44	2.75E+06		31.14	5.30	36	4.77
588.46	5.12E+06		57.93	9.86	37	9.12
589.45	8.08E+06		91.40	15.55	38	14.78
590.47	8.84E+06		100.00	17.02	39	16.59
591.41	5.70E+06		64.42	10.96	40	10.96
599.27	2.54E+05	$[M]^+$	2.87	0.49	31	0.38
603.36	3.04E+05		3.43	0.58	35	0.51
604.31	3.40E+05		3.85	0.65	36	0.59
605.39	8.26E+05		9.35	1.59	37	1.47
606.42	1.33E+06		15.06	2.56	38	2.43
607.41#	1.67E+06	$[M+H]^+$	18.90	3.22	38	3.06
608.49#	1.25E+06		14.10	2.40	39	2.34
609.51	3.52E+05		3.99	0.68	40	0.68
		Total	587.66	100.00	**ΣDoL**	**74.14**

Overlapping mass peaks.

Table A11. Peak assignment and calculation of BPI_i, $BPI_{i(norm)}$ and DoL_i for individual isotopologs of Chl *a* from a non-labeled Arabidopsis plant obtained by FTICR-MS. Beside the predominant $[M+H]^+$ and a small peak of $[M]^+$ at *m/z* ~892, formation of $[M+K]^+$ adduct was observed for Chl *a*. With a resolution of 100,000 at *m/z* 400, mass peaks of isotopologs having similar *m/z* with different Mg isotopes (e.g. ${}^{12}C_{53}{}^{13}C_2H_{73}O_5N_4{}^{24}Mg$, ${}^{12}C_{54}{}^{13}CH_{73}O_5N_4{}^{25}Mg$ and ${}^{12}C_{55}H_{73}O_5N_4{}^{26}Mg$) are overlapping except at *m/z* ~896 where the peak of ${}^{12}C_{54}{}^{13}CH_{73}O_5N_4{}^{26}Mg$ was separated from ${}^{12}C_{52}{}^{13}C_3H_{73}O_5N_4{}^{24}Mg$ and ${}^{12}C_{53}{}^{13}C_2H_{73}O_5N_4{}^{25}Mg$. The BPI_i was calculated for ^{24}Mg-Chl isotopologs based on the natural abundance of Mg isotopes (^{24}Mg 79%; ^{25}Mg 10%; ^{26}Mg 11%). The $[M]^+$ ion was taken into account only at *m/z* ~892. Comparable ΣDoL values were obtained for $[M+H]^+$ and $[M+K]^+$ of ^{24}Mg-Chl. The mass spectrum of this sample is shown in Fig. 7a. *i* gives the number of ^{13}C atom in the molecule.

m/z	**Intensity**	**Δ** (ppm)	**Empirical formula**	**Quasi-molecular ion**	**Calculated BPI_i (^{24}Mg)**	**$BPI_{i\,(norm)}$** (%)	***i***	**DoL_i** (%)
892.53512	188998.7	0.40	${}^{12}C_{55}H_{72}O_5N_4Mg$	$[M]^+$	3.43	1.83	0	0.00
893.5426#	5512974.0	0.02	${}^{12}C_{55}H_{73}O_5N_4Mg$	$[M+H]^+$	100.00	53.43	0	0.00
894.54574#	4567444.5	-0.23	${}^{12}C_{54}{}^{13}CH_{73}O_5N_4Mg$		70.19	37.50	1	0.68
895.55073#	1611089.9	1.60	${}^{12}C_{53}{}^{13}C_2H_{73}O_5N_4Mg$		6.41	3.43	2	0.12
896.54215	410161.3	-1.50	${}^{12}C_{54}{}^{13}CH_{73}O_5N_4{}^{26}Mg$		(^{26}Mg)		(1)	
896.55531#	338695.9	2.97	${}^{12}C_{52}{}^{13}C_3H_{73}O_5N_4Mg$		5.33	2.85	3	0.16
897.54643#	186399.4	-0.47	${}^{12}C_{53}{}^{13}C_2H_{73}O_5N_4{}^{26}Mg$		1.81	0.97	4	0.07
				Total	181.47	100.00	**ΣDoL**	**1.03**
931.49912	613330.3	0.69	${}^{12}C_{55}H_{72}O_5N_4KMg$	$[M+K]^+$	11.13	56.43	0	0.00
932.50246#	470547.5	0.68	${}^{12}C_{54}{}^{13}CH_{72}O_5N_4KMg$		7.13	36.16	1	0.66
933.50798#	173129.5	3.00	${}^{12}C_{53}{}^{13}C_2H_{72}O_5N_4KMg$		0.69	3.49	2	0.13
934.50071#	76748.5	1.43	${}^{12}C_{54}{}^{13}CH_{72}O_5N_4K{}^{26}Mg$		0.31	1.57	3	0.09
935.50316#	33227.2	0.46	${}^{12}C_{53}{}^{13}C_2H_{72}O_5N_4K{}^{26}Mg$		0.47	2.36	4	0.17
				Total	19.72	100.00	**ΣDoL**	**1.04**

Overlapping mass peaks.

Table A12. Peak assignment and calculation of BPI_i, $BPI_{i(norm)}$ and DoL_i for individual isotopologs of Chl *a* from a non-labeled Arabidopsis plant obtained by TQ-MS. Formation of $[M+H]^+$ and $[M+K]^+$ was also observed with TQ-MS, while $[M]^+$ at *m/z* ~892 was hardly detected. Mass peaks of isotopologs having similar *m/z* values with different Mg isotopes are overlapping. The BPI_i was calculated for ^{24}Mg-Chl isotopologs based on the natural abundance of Mg isotopes. TQ-MS also gave comparable ΣDoL values for $[M+H]^+$ and $[M+K]^+$, although both are higher than the corresponding values of FTICR-MS (cf. Additional file 2; Table A11). The mass spectrum of this sample is shown in Fig. 7b. *i* gives the number of ^{13}C atom in the molecule.

m/z	**Intensity**	**Quasi-molecular ion**	**Calculated BPI_i (^{24}Mg)**	**$BPI_{i\,(norm)}$ (%)**	***i***	**DoL_i (%)**
893.49	7.59E+07	$[M+H]^+$	98.01	43.18	0	0.00
894.46#	7.74E+07		87.58	38.59	1	0.70
895.45#	4.54E+07		33.94	14.96	2	0.54
896.46#	1.82E+07		7.03	3.10	3	0.17
897.5#	4.66E+06		0.39	0.17	4	0.01
		Total	226.96	100.00	**ΣDoL**	**1.43**
931.45	1.68E+07	$[M+K]^+$	21.74	39.89	0	0.00
932.4#	1.73E+07		19.53	35.83	1	0.65
933.4#	1.18E+07		9.67	17.75	2	0.65
934.4#	5.36E+06		2.98	5.46	3	0.30
935.42#	1.79E+06		0.59	1.07	4	0.08
		Total	19.72	100.00	**ΣDoL**	**1.67**

Overlapping mass peaks.

Table A13. Peak assignment and calculation of BPI_i, $BPI_{i(norm)}$ and DoL_i for individual isotopologs of Chl *a* from a ^{13}C-labeled Arabidopsis plant obtained by FTICR-MS. As seen in the non-labeled sample (Additional file 2; Table A11), mass peaks of isotopologs having similar *m/z* values with different Mg isotopes are overlapping except at *m/z* ~896. Non-labeled $[M+K]^+$ and ^{13}C-labeled $[M+H]^+$ are separated at *m/z* 931–933. The BPI_i was calculated for $[M+H]^+$ ion of ^{24}Mg-Chl isotopologs (but including $[M]^+$ at *m/z* ~892) based on the natural abundance of Mg isotopes. White and shaded cells denote non-labeled and ^{13}C-labeled isotopologs of Chl *a*, respectively. The mass spectrum of this sample is shown in Fig. 7c. Mass peaks of ^{13}C-labeled $[M+K]^+$ are not included in this table. *i* gives the number of ^{13}C atom in the molecule.

m/z	Intensity	Δ (ppm)	Empirical formula	Quasi-molecular ion	Calculated BPI_i (^{24}Mg)	$BPI_{i\,(norm)}$ (%)	*i*	DoL_i (%)
892.53347	28653.7	-1.45	$^{12}C_{55}H_{72}O_5N_4Mg$	$[M]^+$	3.13	0.37	0	0.00
893.5427#	879791.1	0.13	$^{12}C_{55}H_{73}O_5N_4Mg$	$[M+H]^+$	95.59	11.28	0	0.00
894.54587#	739793.0	-0.08	$^{12}C_{54}{}^{13}CH_{73}O_5N_4Mg$		68.17	8.05	1	0.15
895.551#	244971.4	1.90	$^{12}C_{53}{}^{13}C_2\,H_{73}O_5N_4Mg$		4.79	0.57	2	0.02
896.53977	48850.9	-4.16	$^{12}C_{54}{}^{13}CH_{73}O_5N_4{}^{26}Mg$		(^{26}Mg)		(1)	
896.55614#	70429.6	3.89	$^{12}C_{52}{}^{13}C_3\,H_{73}O_5N_4Mg$		1.75	0.21	3	0.01
897.54475#	22296.8	-2.34	$^{12}C_{53}{}^{13}C_2H_{73}O_5N_4{}^{26}Mg$		1.54	0.18	4	0.01
931.4993*	96412.2	0.89	$^{12}C_{55}H_{72}O_5N_4KMg$	$[M+K]^+$			0	
932.50229*#	68702.4	0.49	$^{12}C_{54}{}^{13}CH_{72}O_5N_4KMg$				1	
933,5063*#	32346.5	1.20	$^{12}C_{53}{}^{13}C_2H_{72}O_5N_4KMg$				2	
906.58752#	12780.4	1.46	$^{12}C_{42}{}^{13}C_{13}H_{73}O_5N_4Mg$	$[M+H]^+$	1.39	0.16	13	0.04
907.58888#	12505.9	-0.75	$^{12}C_{41}{}^{13}C_{14}H_{73}O_5N_4Mg$		1.19	0.14	14	0.04
908.59244#	25631.8	-0.52	$^{12}C_{40}{}^{13}C_{15}H_{73}O_5N_4Mg$		2.45	0.29	15	0.08
909.59635#	11707.4	0.09	$^{12}C_{39}{}^{13}C_{16}H_{73}O_5N_4Mg$		0.80	0.09	16	0.03
910.60124#	34539.2	1.78	$^{12}C_{38}{}^{13}C_{17}H_{73}O_5N_4Mg$		3.33	0.39	17	0.12
911.60324#	63008.9	0.29	$^{12}C_{37}{}^{13}C_{18}H_{73}O_5N_4Mg$		6.34	0.74	18	0.24
912.60728#	64216.5	1.04	$^{12}C_{36}{}^{13}C_{19}H_{73}O_5N_4Mg$		5.74	0.67	19	0.23
913.61032#	94490.8	0.69	$^{12}C_{35}{}^{13}C_{20}H_{73}O_5N_4Mg$		8.70	1.02	20	0.37
914.61357#	38101.7	0.58	$^{12}C_{34}{}^{13}C_{21}H_{73}O_5N_4Mg$		2.26	0.26	21	0.10
915.61827#	31007.6	2.05	$^{12}C_{33}{}^{13}C_{22}H_{73}O_5N_4Mg$		1.89	0.22	22	0.09
916.62315#	16512.2	3.71	$^{12}C_{32}{}^{13}C_{23}H_{73}O_5N_4Mg$		1.25	0.15	23	0.06
917.62441#	28883.8	1.42	$^{12}C_{31}{}^{13}C_{24}H_{73}O_5N_4Mg$		2.73	0.32	24	0.14
918.62727#	29492.6	0.88	$^{12}C_{30}{}^{13}C_{25}H_{73}O_5N_4Mg$		2.70	0.32	25	0.14
919.63033#	25200.2	0.56	$^{12}C_{29}{}^{13}C_{26}H_{73}O_5N_4Mg$		2.03	0.24	26	0.11
920.63398#	39680.3	0.88	$^{12}C_{28}{}^{13}C_{27}H_{73}O_5N_4Mg$		3.70	0.43	27	0.21
921.63649#	45717.7	-0.03	$^{12}C_{27}{}^{13}C_{28}H_{73}O_5N_4Mg$		4.24	0.50	28	0.25
922.64125#	38429.8	1.49	$^{12}C_{26}{}^{13}C_{29}H_{73}O_5N_4Mg$		3.14	0.37	29	0.19
923.64366#	27097.2	0.46	$^{12}C_{25}C_{30}H_{73}O_5N_4Mg$		1.97	0.23	30	0.13
924.64754#	31882.0	1.03	$^{12}C_{24}{}^{13}C_{31}H_{73}O_5N_4Mg$		2.79	0.33	31	0.18
925.65088#	47732.9	1.02	$^{12}C_{23}{}^{13}C_{32}H_{73}O_5N_4Mg$		4.58	0.54	32	0.31
926.65358#	44747.9	0.30	$^{12}C_{22}{}^{13}C_{33}H_{73}O_5N_4Mg$		3.91	0.46	33	0.28
927.65647#	46441.1	-0.20	$^{12}C_{21}{}^{13}C_{34}H_{73}O_5N_4Mg$		3.93	0.46	34	0.29
928.66028#	49235.3	0.30	$^{12}C_{20}{}^{13}C_{35}H_{73}O_5N_4Mg$		4.33	0.51	35	0.32
929.66457#	45550.4	1.30	$^{12}C_{19}{}^{13}C_{36}H_{73}O_5N_4Mg$		3.87	0.45	36	0.30
930.66781#	56167.2	1.18	$^{12}C_{18}{}^{13}C_{37}H_{73}O_5N_4Mg$		5.03	0.59	37	0.40
931.67188*#	55627.6	1.94	$^{12}C_{17}{}^{13}C_{38}H_{73}O_5N_4Mg$		4.89	0.57	38	0.40
932.67432*#	78952.0	0.96	$^{12}C_{16}{}^{13}C_{39}H_{73}O_5N_4Mg$		7.29	0.86	39	0.61
933.67818*#	79418.9	1.50	$^{12}C_{15}{}^{13}C_{40}H_{73}O_5N_4Mg$		7.06	0.83	40	0.60
934.6808#	99179.4	0.71	$^{12}C_{14}{}^{13}C_{41}H_{73}O_5N_4Mg$		8.91	1.05	41	0.78
935.68435#	105362.9	0.92	$^{12}C_{13}{}^{13}C_{42}H_{73}O_5N_4Mg$		9.38	1.10	42	0.84
936.68784#	163363.7	1.06	$^{12}C_{12}{}^{13}C_{43}H_{73}O_5N_4Mg$		15.39	1.81	43	1.41
937.69151#	182465.1	1.40	$^{12}C_{11}{}^{13}C_{44}H_{73}O_5N_4Mg$		16.65	1.97	44	1.56
938.69471#	255304.8	1.23	$^{12}C_{10}{}^{13}C_{45}H_{73}O_5N_4Mg$		23.60	2.77	45	2.27
939.69808#	318775.7	1.24	$^{12}C_9{}^{13}C_{46}H_{73}O_5N_4Mg$		29.47	3.46	46	2.89
940.70115#	381412.0	0.95	$^{12}C_8{}^{13}C_{47}H_{73}O_5N_4Mg$		34.59	4.06	47	3.47
941.70443#	429322.6	0.86	$^{12}C_7{}^{13}C_{48}H_{73}O_5N_4Mg$		38.36	4.50	48	3.93
942.70796#	603606.2	1.05	$^{12}C_6{}^{13}C_{49}H_{73}O_5N_4Mg$		56.18	6.59	49	5.87

943.71125#	694721.1	0.98	$^{12}C_5{}^{13}C_{50}H_{73}O_5N_4Mg$		63.34	7.43	50	6.75
944.71456#	909568.5	0.93	$^{12}C_4{}^{13}C_{51}H_{73}O_5N_4Mg$		83.39	9.78	51	9.07
945.71798#	916611.8	1.00	$^{12}C_3{}^{13}C_{52}H_{73}O_5N_4Mg$		80.62	9.46	52	8.94
946.72122#	816270.6	0.87	$^{12}C_2{}^{13}C_{53}H_{73}O_5N_4Mg$		67.24	7.89	53	7.60
947.7249#	547624.9	1.21	$^{12}C^{13}C_{54}H_{73}O_5N_4Mg$		40.01	4.69	54	4.61
948.7287#	195318.5	1.69	$^{13}C_{55}H_{73}O_5N_4Mg$		6.88	0.81	55	0.81
				Total	852.53	100.00	**ΣDoL**	**67.25**

# Overlapping mass peaks.

* Overlapping mass peaks in TQ-MS that are separated by FTICR-MS.

Table A14. Peak assignment and calculation of BPI$_i$, BPI$_{i(\mathrm{norm})}$ and DoL$_i$ for individual isotopologs of Chl *a* from a ^{13}C-labeled Arabidopsis plant obtained by TQ-MS. The contribution of non-labeled $[M+K]^+$ peaks overlapping with those of ^{13}C-labeled $[M+H]^+$ at *m*/*z* 931–934 was estimated from the intensity of non-labeled $[M+H]^+$ peaks and the ratio between $[M+H]^+$ and $[M+K]^+$ found in the non-labeled sample (see Fig. 7b and Additional file 2; Table A12). The peaks at *m*/*z* 915–917 are presumably overlapping with $[M+Na]^+$ and thus not included in the analysis. Since mass peaks of isotopologs having similar *m*/*z* values with different Mg isotopes are overlapping, BPI$_i$ was calculated for $[M+H]^+$ ion of ^{24}Mg-Chl isotopologs based on the natural abundance of Mg isotopes. White and shaded cells denote non-labeled and ^{13}C-labeled isotopologs of Chl *a*, respectively. The mass spectrum of this sample is shown in Fig. 7d. Peaks of $[M+K]^+$ are not included in this table. *i* gives the number of ^{13}C atom in the molecule.

m/z	**Intensity**	**Quasi-molecular ion**	**Calculated BPI$_i$ (^{24}Mg)**	**BPI$_{i\,(\mathrm{norm})}$ (%)**	***i***	**DoL$_i$ (%)**
893.47#	1.64E+07	$[M+H]^+$	71.73	8.64	0	0.00
894.43#	1.55E+07		58.50	7.05	1	0.13
895.42#	8.91E+06		21.54	2.59	2	0.09
896.43#	3.52E+06		4.50	0.54	3	0.03
907.43#	4.02E+04	$[M+H]^+$	0.18	0.02	14	0.01
908.45#	9.31E+04		0.38	0.05	15	0.01
909.45#	1.55E+05		0.60	0.07	16	0.02
910.46#	5.07E+05		2.08	0.25	17	0.08
911.47#	9.68E+05		3.88	0.47	18	0.15
912.48#	1.71E+06		6.68	0.81	19	0.28
913.49#	1.98E+06		7.27	0.88	20	0.32
914.48#	1.37E+06		4.14	0.50	21	0.19
918.49#	7.99E+05		3.49	0.42	25	0.19
919.5#	6.00E+05		2.18	0.26	26	0.12
920.51#	5.41E+05		1.60	0.19	27	0.09
921.49#	7.21E+05		2.64	0.32	28	0.16
922.52#	7.59E+05		2.76	0.33	29	0.18
923.51#	8.75E+05		3.11	0.37	30	0.20
924.5#	1.01E+06		3.64	0.44	31	0.25
925.51#	4.81E+05		1.21	0.15	32	0.08
926.52#	8.33E+05		2.98	0.36	33	0.22
927.52#	9.90E+05		3.78	0.46	34	0.28
928.52#	1.16E+06		4.18	0.50	35	0.32
929.53#	1.05E+06		3.55	0.43	36	0.28
930.38#	6.75E+05		1.92	0.23	37	0.16
931.45#	8.09E+05		2.80	0.34	38	0.23
932.44#	1.01E+06		3.77	0.45	39	0.32
933.47#	2.08E+06		8.22	0.99	40	0.72
934.5#	2.71E+06		10.29	1.24	41	0.92
935.53#	3.54E+06		13.00	1.57	42	1.20
936.55#	3.90E+06		13.96	1.68	43	1.31
937.56#	4.72E+06		17.03	2.05	44	1.64
938.56#	5.88E+06		21.60	2.60	45	2.13
939.56#	7.24E+06		26.50	3.19	46	2.67
940.57#	9.06E+06		33.20	4.00	47	3.42
941.57#	1.11E+07		40.68	4.90	48	4.28
942.57#	1.38E+07		50.51	6.08	49	5.42
943.58#	1.68E+07		61.11	7.36	50	6.69
944.59#	2.02E+07		73.43	8.84	51	8.20
945.59#	2.27E+07		81.53	9.82	52	9.28
946.59#	2.29E+07		79.44	9.57	53	9.22
947.6#	1.77E+07		55.99	6.74	54	6.62
948.59#	8.42E+06		18.64	2.25	55	2.25
		Total	830.24	100.00	**ΣDoL**	**70.37**

Overlapping mass peaks.

Table A15. Peak assignment and calculation of BPI_i, $BPI_{i(norm)}$ and DoL_i for individual isotopologs of Chl *b* from a non-labeled Arabidopsis plant obtained by FTICR-MS. Only $[M+H]^+$ and a small peak of $[M]^+$ (*m*/*z* ~906) were found for Chl *b* by FTICR-MS. As seen for Chl *a* (Additional file 2; Tables A11, A13), mass peaks of isotopologs having similar *m*/*z* values with different Mg isotopes are overlapping also for Chl *b*. The BPI_i was calculated for ^{24}Mg-Chl isotopologs based on the natural abundance of Mg isotopes. The mass spectrum of this sample is shown in Fig. 8a. *i* gives the number of ^{13}C atom in the molecule.

m/z	**Intensity**	**Δ** (ppm)	**Empirical formula**	**Quasi-molecular ion**	**Calculated BPI_i (^{24}Mg)**	**$BPI_{i\,(norm)}$** (%)	***i***	**DoL_i** (%)
906.51498	40735.6	1.05	$^{12}C_{55}H_{70}O_6N_4Mg$	$[M]^+$	2.07	1.15	0	0.00
907.52208#	1966119.3	0.25	$^{12}C_{55}H_{71}O_6N_4Mg$	$[M+H]^+$	100.00	55.61	0	0.00
908.5253#	1656528.5	0.10	$^{12}C_{54}{}^{13}CH_{71}O_6N_4Mg$		71.59	39.81	1	0.72
909.53018#	573470.5	1.78	$^{12}C_{53}{}^{13}C_2H_{71}O_6N_4Mg$		6.17	3.43	2	0.12
				Total	179.83	100.00	**ΣDoL**	**0.85**

Overlapping mass peaks.

Table A16. Peak assignment and calculation of BPI_i, $BPI_{i(norm)}$ and DoL_i for individual isotopologs of Chl *b* from a non-labeled Arabidopsis plant obtained by TQ-MS. As was the case for Chl *a*, formation of $[M+H]^+$ and $[M+K]^+$ was observed with TQ-MS, while $[M]^+$ at *m*/*z* ~906 was hardly detected. Mass peaks of isotopologs having similar *m*/*z* values with different Mg isotopes are overlapping. The BPI_i was calculated for ^{24}Mg-Chl isotopologs based on the natural abundance of Mg isotopes. The ΣDoL values of $[M+H]^+$ and $[M+K]^+$ are very similar to the corresponding values of Chl *a* (Additional file 2; Table A12). The mass spectrum of this sample is shown in Fig. 8b. *i* gives the number of ^{13}C atom in the molecule.

m/z	**Intensity**	**Quasi-molecular ion**	**Calculated BPI_i (^{24}Mg)**	**$BPI_{i\,(norm)}$** (%)	***i***	**DoL_i** (%)
907.46	2.94E+07	$[M+H]^+$	100.00	43.84	0	0.00
908.42#	2.92E+07		86.86	38.08	1	0.69
909.42#	1.72E+07		33.72	14.78	2	0.54
910.43#	6.94E+06		7.26	3.18	3	0.17
911.47#	1.73E+06		0.29	0.13	4	0.01
		Total	228.13	100.00	**ΣDoL**	**1.41**
945.42	1.75E+07	$[M+K]^+$	59.67	40.80	0	0.00
946.37#	1.74E+07		51.81	35.42	1	0.64
947.37#	1.19E+07		25.72	17.59	2	0.64
948.39#	5.28E+06		7.49	5.12	3	0.28
949.41#	1.79E+06		1.58	1.08	4	0.08
		Total	146.27	100.00	**ΣDoL**	**1.64**

Overlapping mass peaks.

Table A17. Peak assignment and calculation of BPI_i, $BPI_{i(norm)}$ and DoL_i for individual isotopologs of Chl *b* from a ^{13}C-labeled Arabidopsis plant obtained by FTICR-MS. Mass peaks of isotopologs having similar *m/z* values with different Mg isotopes are overlapping. The BPI_i was calculated for $[M+H]^+$ ion of ^{24}Mg-Chl isotopologs (but including $[M]^+$ at *m/z* ~906) based on the natural abundance of Mg isotopes. No $[M+K]^+$ adduct of Chl *b* was observed in FTICR-MS data. White and shaded cells denote non-labeled and ^{13}C-labeled isotopologs of Chl *b*, respectively. The mass spectrum of this sample is shown in Fig. 8c. *i* gives the number of ^{13}C atom in the molecule.

m/z	Intensity	Δ (ppm)	Empirical formula	Quasi-molecular ion	Calculated BPI_i (^{24}Mg)	$BPI_{i\,(norm)}$ (%)	*i*	DoL_i (%)
906.51505	9457.2	1.12	$^{12}C_{55}H_{71}O_6N_4Mg$	$[M]^+$	1.66	0.33	0	0.00
907.5219#	571323.9	0.05	$^{12}C_{55}H_{71}O_6N_4Mg$	$[M+H]^+$	100.00	19.83	0	0.00
908.52501#	423242.5	-0.22	$^{12}C_{54}{}^{13}CH_{71}O_6N_4Mg$		61.42	12.18	1	0.22
909.53015#	144275.2	1.74	$^{12}C_{53}{}^{13}C_2H_{71}O_6N_4Mg$		3.54	0.70	2	0.03
925.58233#	23342	0.10	$^{12}C_{37}{}^{13}C_{18}H_{71}O_6N_4Mg$	$[M+H]^+$	4.09	0.81	18	0.27
926.58599#	34546.7	0.43	$^{12}C_{36}{}^{13}C_{19}H_{71}O_6N_4Mg$		5.53	1.10	19	0.38
927.58994#	32982.2	1.07	$^{12}C_{35}{}^{13}C_{20}H_{71}O_6N_4Mg$		4.50	0.89	20	0.32
928.59445#	20522.9	2.31	$^{12}C_{34}{}^{13}C_{21}H_{71}O_6N_4Mg$		2.25	0.45	21	0.17
929.59733#	19711.7	1.80	$^{12}C_{33}{}^{13}C_{22}H_{71}O_6N_4Mg$		2.54	0.50	22	0.20
930.59968#	8768.6	0.71	$^{12}C_{32}{}^{13}C_{23}H_{71}O_6N_4Mg$		0.90	0.18	23	0.07
931.60378#	9605.3	1.52	$^{12}C_{31}{}^{13}C_{24}H_{71}O_6N_4Mg$		1.21	0.24	24	0.10
932.60478#	6307.8	-1.01	$^{12}C_{30}{}^{13}C_{25}H_{71}O_6N_4Mg$		0.82	0.16	25	0.07
933.61026#	16765.6	1.26	$^{12}C_{29}{}^{13}C_{26}H_{71}O_6N_4Mg$		2.66	0.53	26	0.25
934.61277#	14548.1	0.36	$^{12}C_{28}{}^{13}C_{27}H_{71}O_6N_4Mg$		2.10	0.42	27	0.20
935.61648#	11249.1	0.74	$^{12}C_{27}{}^{13}C_{28}H_{71}O_6N_4Mg$		1.33	0.26	28	0.13
936.62237#	11720.1	3.45	$^{12}C_{26}{}^{13}C_{29}H_{71}O_6N_4Mg$		1.59	0.32	29	0.17
937.62423#	20526.4	1.85	$^{12}C_{25}{}^{13}C_{30}H_{71}O_6N_4Mg$		3.20	0.64	30	0.35
938.6266#	18723.3	0.79	$^{12}C_{24}{}^{13}C_{31}H_{71}O_6N_4Mg$		2.65	0.53	31	0.30
939.62834#	17492.5	-0.92	$^{12}C_{23}{}^{13}C_{32}H_{71}O_6N_4Mg$		2.28	0.45	32	0.26
940.63257#	15451.3	0.01	$^{12}C_{22}{}^{13}C_{33}H_{71}O_6N_4Mg$		2.04	0.40	33	0.24
941.6366#	26773.4	0.72	$^{12}C_{21}{}^{13}C_{34}H_{71}O_6N_4Mg$		4.11	0.82	34	0.50
942.64006#	20861.7	0.83	$^{12}C_{20}{}^{13}C_{35}H_{71}O_6N_4Mg$		2.84	0.56	35	0.36
943.64502#	31380.9	2.53	$^{12}C_{19}{}^{13}C_{36}H_{71}O_6N_4Mg$		4.56	0.90	36	0.59
944.64784#	23686.4	1.97	$^{12}C_{18}{}^{13}C_{37}H_{71}O_6N_4Mg$		3.18	0.63	37	0.42
945.65081#	29133.1	1.56	$^{12}C_{17}{}^{13}C_{38}H_{71}O_6N_4Mg$		4.06	0.81	38	0.56
946.65312#	42466.8	0.46	$^{12}C_{16}{}^{13}C_{39}H_{71}O_6N_4Mg$		6.47	1.28	39	0.91
947.65752#	26799.9	1.56	$^{12}C_{15}{}^{13}C_{40}H_{71}O_6N_4Mg$		3.30	0.66	40	0.48
948.6607#	51677.1	1.37	$^{12}C_{14}{}^{13}C_{41}H_{71}O_6N_4Mg$		7.73	1.53	41	1.14
949.66314#	56303.4	0.41	$^{12}C_{13}{}^{13}C_{42}H_{71}O_6N_4Mg$		8.41	1.67	42	1.27
950.66677#	77378.9	0.69	$^{12}C_{12}{}^{13}C_{43}H_{71}O_6N_4Mg$		11.40	2.26	43	1.77
951.67022#	76532.3	0.80	$^{12}C_{11}{}^{13}C_{44}H_{71}O_6N_4Mg$		10.78	2.14	44	1.71
952.67356#	106072.2	0.78	$^{12}C_{10}{}^{13}C_{45}H_{71}O_6N_4Mg$		15.62	3.10	45	2.53
953.67669#	117753.7	0.54	$^{12}C_9{}^{13}C_{46}H_{71}O_6N_4Mg$		17.13	3.40	46	2.84
954.68039#	139073.4	0.90	$^{12}C_8{}^{13}C_{47}H_{71}O_6N_4Mg$		19.99	3.97	47	3.39
955.68375#	170621.5	0.91	$^{12}C_7{}^{13}C_{48}H_{71}O_6N_4Mg$		25.02	4.96	48	4.33
956.68695#	182718.1	0.74	$^{12}C_6{}^{13}C_{49}H_{71}O_6N_4Mg$		26.03	5.16	49	4.60
957.6904#	201488.3	0.84	$^{12}C_5{}^{13}C_{50}H_{71}O_6N_4Mg$		28.49	5.65	50	5.14
958.6936#	224523.6	0.68	$^{12}C_4{}^{13}C_{51}H_{71}O_6N_4Mg$		32.07	6.36	51	5.90
959.69668#	232619.5	0.39	$^{12}C_3{}^{13}C_{52}H_{71}O_6N_4Mg$		32.69	6.48	52	6.13
960.70035#	182189.4	0.72	$^{12}C_2{}^{13}C_{53}H_{71}O_6N_4Mg$		23.28	4.62	53	4.45
961.70377#	97929.7	0.79	$^{12}C{}^{13}C_{54}H_{71}O_6N_4Mg$		9.64	1.91	54	1.88
962.7091#	31946.7	2.84	$^{13}C_{55}H_{71}O_6N_4Mg$		1.12	0.22	55	0.22
				Total	504.24	100.00	**ΣDoL**	**54.86**

Overlapping mass peaks.

Table A18. Peak assignment and calculation of BPI_i, $BPI_{i(norm)}$ and DoL_i for individual isotopologs of Chl *b* from a ^{13}C-labeled Arabidopsis plant obtained by TQ-MS. The contribution of non-labeled $[M+K]^+$ overlapping with ^{13}C-labeled $[M+H]^+$ at *m*/*z* 945–949 was estimated from the intensity of non-labeled $[M+H]^+$ peaks and the ratio between $[M+H]^+$ and $[M+K]^+$ found in the non-labeled sample (see Fig. 8b and Additional file 2; Table A16). Since mass peaks of isotopologs having similar *m*/*z* values with different Mg isotopes are overlapping, BPI_i was calculated for $[M+H]^+$ ion of ^{24}Mg-Chl isotopologs based on the natural abundance of Mg isotopes. The peaks at *m*/*z* 929–931 are presumably overlapping with $[M+Na]^+$ and thus not included in the analysis. White and shaded cells denote non-labeled and ^{13}C-labeled isotopologs of Chl *b*, respectively. The mass spectrum of this sample is shown in Fig. 8d. *i* gives the number of ^{13}C atom in the molecule.

m/z	**Intensity**	**Quasi-molecular ion**	**Calculated BPI_i (^{24}Mg)**	**$BPI_{i\,(norm)}$ (%)**	***i***	**DoL_i (%)**
907.45#	1.04E+07	$[M+H]^+$	100.00	15.88	0	0.00
908.4#	9.46E+06		78.66	12.49	1	0.23
909.4#	5.40E+06		28.26	4.49	2	0.16
910.4#	2.01E+06		4.84	0.77	3	0.04
932.47#	3.54E+05	$[M+H]^+$	3.42	0.54	25	0.25
933.48#	3.40E+05		2.85	0.45	26	0.21
934.48#	3.28E+05		2.32	0.37	27	0.18
935.5#	3.46E+05		2.65	0.42	28	0.21
936.48#	3.14E+05		2.37	0.38	29	0.20
937.48#	3.30E+05		2.51	0.40	30	0.22
938.5#	3.87E+05		3.09	0.49	31	0.28
939.5#	3.81E+05		2.93	0.47	32	0.27
940.49#	4.11E+05		3.16	0.50	33	0.30
941.52#	3.41E+05		2.49	0.39	34	0.24
942.51#	4.01E+05		3.12	0.50	35	0.32
943.51#	3.07E+05		2.22	0.35	36	0.23
947.38#	1.10E+06		10.59	1.68	40	1.22
948.44#	1.52E+06		13.36	2.12	41	1.58
949.48#	1.68E+06		13.08	2.08	42	1.59
950.51#	1.87E+06		14.52	2.31	43	1.80
951.53#	1.94E+06		15.08	2.39	44	1.92
952.53#	2.26E+06		17.89	2.84	45	2.32
953.54#	2.65E+06		21.20	3.37	46	2.82
954.54#	3.11E+06		24.81	3.94	47	3.37
955.55#	3.64E+06		29.01	4.61	48	4.02
956.56#	4.18E+06		33.19	5.27	49	4.69
957.55#	4.60E+06		36.14	5.74	50	5.22
958.55#	5.20E+06		41.02	6.51	51	6.04
959.56#	5.39E+06		41.82	6.64	52	6.28
960.56#	5.09E+06		38.15	6.06	53	5.84
961.57#	3.69E+06		25.00	3.97	54	3.90
962.58#	1.92E+06		10.04	1.59	55	1.59
		Total	629.79	100.00	**ΣDoL**	**57.54**

Overlapping mass peaks.
